# An Unexpectedly High Number of New *Sutorius* (Boletaceae) Species From Northern and Northeastern Thailand

**DOI:** 10.3389/fmicb.2021.643505

**Published:** 2021-04-12

**Authors:** Santhiti Vadthanarat, Roy E. Halling, Mario Amalfi, Saisamorn Lumyong, Olivier Raspé

**Affiliations:** ^1^Department of Biology, Faculty of Science, Chiang Mai University, Chiang Mai, Thailand; ^2^Research Center of Microbial Diversity and Sustainable Utilization, Faculty of Science, Chiang Mai University, Chiang Mai, Thailand; ^3^Institute of Systematic Botany, New York Botanical Garden, Bronx, NY, United States; ^4^Botanic Garden Meise, Meise, Belgium; ^5^Fédération Wallonie-Bruxelles, Service Général de l’Enseignement Universitaire et de la Recherche Scientifique, Bruxelles, Belgium; ^6^Academy of Science, The Royal Society of Thailand, Bangkok, Thailand; ^7^School of Science, Mae Fah Luang University, Chiang Rai, Thailand; ^8^Center of Excellence in Fungal Research, Mae Fah Luang University, Chiang Rai, Thailand

**Keywords:** *Boletales*, eight new taxa, multigene phylogeny, mushrooms, *Pulveroboletus* group, taxonomy

## Abstract

*Sutorius* is a poroid genus in Boletaceae that typically has chocolate brown to reddish brown or purplish brown basidiomata with a finely scaly stipe and produces a reddish brown spore deposit. During the survey on diversity of boletes in Northern and Northeastern Thailand, several *Sutorius* collections were obtained. Combined evidence from morphology and phylogenetic analyses of a combined three-gene data set (*atp*6, *tef*1 and *rpb*2) of the *Sutorius* collections along with selected Boletaceae in the *Pulveroboletus* group indicated that Thai collections represent seven new *Sutorius* species. The analyses also indicated that *Tylopilus maculatoides* belongs in *Sutorius*. Therefore, the transfer of *T. maculatoides* to *Sutorius* is proposed. Full descriptions and illustrations of the seven new species and *S. maculatoides* are presented in this study. With the seven new species and the new combination, eight of the eleven described *Sutorius* species are known to occur in Northern and Northeastern Thailand, whereas only one species is known from each of two continents, the Americas and Australia.

## Introduction

*Sutorius* Halling, Nuhn and N.A. Fechner was established in 2012 to accommodate two bolete species, *Boletus eximius* Peck [current name = *Sutorius eximius* (Peck) Halling, Nuhn and Osmundson] and *Leccinum australiense* Bougher and Thiers [current name = *Sutorius australiensis* (Bougher and Thiers) Halling and N.A. Fechner] (Halling et al., 2012). Typical characteristics of *Sutorius* are stipitate–pileate basidiomata with poroid hymenophore, chocolate to reddish brown or purplish brown basidiomata with finely scaly stipe, and a reddish brown spore deposit. The original publication of *Sutorius* also reported two *Sutorius* specimens originating from Chiang Mai Province, in Northern Thailand. One of them was morphologically identified as *S*. *eximius*, whereas another was phylogenetically recognized as a different species from *S*. *eximius* and *S*. *australiensis*. However, the latter was not described as a new species at that time (Halling et al., 2012). In 2016, Wu et al., recombined several bolete species (e.g., *Boletus brunneissimus* W.F. Chiu; *B*. *hainanensis* T.H. Li and M. Zang; *B*. *luridiformis* Rostk.; *B*. *obscureumbrinus* Hongo; *B*. *tomentulosus* M. Zang W.P. Liu and M.R. Hu) into the genus *Sutorius* based on their phylogenetic inferences. They also described several new *Sutorius* species from China (e.g., *S*. *ferrugineus* G. Wu, F. Li and Zhu L. Yang; *S*. *ferrugineus* G. Wu, F. Li and Zhu L. Yang; *S*. *rubriporus* G. Wu and Zhu L. Yang). Later, Chai et al. (2019) restudied the phylogenetic relationships of several Boletaceae specimens from subtropical and tropical China, reinstated the genus *Neoboletus* Gelardi, Simonini and Vizzini and recombined all *Sutorius* species described in Wu et al. (2016) to be into this latter. Chai et al. (2019) also newly described a third species of *Sutorius*, *Sutorius subrufus* N.K. Zeng, H. Chai and S. Jiang from Southern China. Moreover, the phylogeny in that study suggested an additional five undescribed *Sutorius* species clustering in 10 independent linages (three described species included). That inference also indicated the exemplars identified as *S*. *eximius*, originating from several different countries (China, Costa Rica, Indonesia, and United States), were in a polytomic lineage (Chai et al., 2019). So far, the distribution of *Sutorius* has been recorded to occur in several regions including the Americas, Africa, Asia, and Australia (Singer, 1947; Smith and Thiers, 1971; Fulgenzi et al., 2007; Chandrasrikul et al., 2011; Halling et al., 2012; Chai et al., 2019). At present, only three species have been placed in the genus (Halling et al., 2012; Chai et al., 2019).

In Thailand, there were a few reports of *Sutorius* species, originally identified as *Tylopilus eximius* (Peck) Singer (current name = *S*. *eximius*) (Chantorn et al., 2007; Chandrasrikul et al., 2008; Vadthanarat, 2009; Vadthanarat et al., 2010). However, the identifications in those reports were based on morphological evidence only, and further phylogenetic studies are needed to confirm those identifications. One Asian species of Boletaceae, *Tylopilus maculatoides* E. Horak, also has similar morphological characters as *Sutorius*. That name was proposed for *Boletus maculatus* Corner non-Raddi based on morphological evidence (Horak, 2011). However, the species has not been included in a phylogenetic context until now (see below).

In this study, morphological and phylogenetic analyses of several *Sutorius* collections, including *T. maculatoides* collections, inferred seven new *Sutorius* species among the Thai collections. Moreover, the phylogram also showed that *T. maculatoides* clustered within the *Sutorius* clade with high support. Consequently, the new combination *Sutorius maculatoides* is proposed. Their phylogenetic affinities and reports of other *Sutorius* species from Thailand or elsewhere are critically discussed.

## Materials and Methods

### Specimen Collection

Fresh basidiomata of *Sutorius* were collected in Chiang Mai Province in the North and Ubon Ratchathani Province, Northeastern Thailand, in 2012 to 2018. They were photographed in the field and then wrapped in aluminum foil for later description in the laboratory. The specimens were then dried after the morphological description in an electric drier at 45°C–50°C. Examined specimens were deposited at Department of National Parks, Wildlife and Plant Conservation, Bangkok, Thailand (BKF), Chiang Mai University, Chiang Mai, Thailand (CMUB), and Mae Fah Luang University, Chiang Rai, Thailand (MFLU) with duplicates in Botanic Garden Meise, Belgium (BR).

### Morphological Studies

Macroscopic descriptions were made based on the detailed field notes and photographs of fresh basidiomata. Color codes are based on Kornerup and Wanscher (1978). Macrochemical reactions (color reactions) were observed using aqueous solutions of 10% KOH, 28–30% NH_4_OH, and Melzer reagent. Microscopic structures were observed from dried specimens and rehydrated in 5% potassium hydroxide or 1% ammoniacal Congo red. For dimensions of microscopic features, a minimum of 50 basidiospores (20 for other structures) were randomly measured at 1,000 × using a calibrated ocular micrometer on an Olympus CX31 compound microscope. The notations “[*n*/*m*/*p*]” show the number of basidiospores “*n*” measured from the number of basidiomata “*m*” of the number of collections “*p*.” Dimensions of microscopic structure are presented in the following (*a*–) *b*–*c*–*d* (–*e*), in which “*c*” contains an average, “*b*” is the 5^th^ percentile, “*d*” is the 95^th^ percentile, and extreme values “*a*” and “*e*” are shown in parentheses. *Q* is the length/width ratio. Sections of the pileipellis were cut radially, perpendicularly to the surface halfway between the disc and margin of pileus. Sections of stipitipellis or the squamules on the stipe were taken halfway along the stipe length (Li et al., 2011; Hosen et al., 2013; Li et al., 2014; Zhu et al., 2015). All microscopic features were drawn free hand using an Olympus Camera Lucida model U-DA.

### DNA Extraction, Polymerase Chain Reaction Amplification, and DNA Sequencing

Genomic DNA was extracted from fresh tissue preserved in CTAB or approximately 10–15 mg of dried tissue using a CTAB isolation procedure adapted from Doyle and Doyle (1990). Parts of three protein-coding genes, *atp*6, *tef*1, and *rpb*2, were amplified by polymerase chain reaction (PCR) and sequenced. ITS-5.8 region of the nuclear ribosomal DNA was not sequenced because in Boletaceae, ITS commonly shows high levels of intraindividual polymorphisms for indels, thus mostly preventing straightforward phylogenetic analysis and moreover often making cloning necessary to obtain a sequence. The selected protein-coding genes have shown multiple times their usefulness for investigating phylogenetic relationships, at both infrageneric and suprageneric levels (e.g., Wu et al., 2016; Vadthanarat et al., 2019). For the amplification of *atp*6, ATP6-1M40F and ATP6-2M primers were used, with following the protocol and PCR program in Raspé et al. (2016). The primers EF1-983F and EF1-2218R (Rehner and Buckley, 2005) were used to amplify *tef*1 gene, and bRPB2-6F and bRPB2-7.1R primers (Matheny, 2005) were used to amplify *rpb*2 gene. PCR products were purified by adding 1 U of exonuclease I and 0.5 U FastAP alkaline phosphatase (Thermo Scientific, St. Leon-Rot, Germany) and incubating at 37°C for 1 h, followed by inactivation at 80°C for 15 min. Sequencing was performed by Macrogen Inc. (Korea and the Netherlands) with PCR primers, except for *atp*6, for which universal primers M13F-pUC(-40) and M13F(-20) were used; for *tef*1, additional sequencing was performed with the two internal primers, EF1-1577F and EF1-1567R (Rehner and Buckley, 2005).

### Alignment and Phylogeny Inference

The sequences were assembled in GENEIOUS Pro v. 6.0.6 (Biomatters). All nucleotide sequences, including nucleotide sequences obtained from GenBank, were aligned using MAFFT version 7 (Katoh and Standley, 2013) on the server accessed at http://mafft.cbrE.jp/alignment/server/.

Before combining the data partitions, topological incongruence between the datasets was assessed using maximum likelihood (ML) and Bayesian inference (BI) separately on each of the four character sets, *atp*6, *tef*1 exons, *rpb*2 exons, and the three introns of *tef*1 + the intron of *rpb*2. Paired trees were examined for conflicts involving only nodes with ML bootstrap (BS) > 75% and Bayesian posterior probabilities (PPs) > 0.95 (Mason-Gamer and Kellogg, 1996; Lutzoni et al., 2004; Reeb et al., 2004). A conflict was assumed to be significant if two different relationships for the same set of taxa (one being monophyletic and the other non-monophyletic) were observed in rival trees. For ML, phylogenetic inference was performed using RAxML (Stamatakis, 2006) on the CIPRES web portal (RAxML-HPC2 on XSEDE; Miller et al., 2009), using the GTRCAT model with 25 categories. Three *Butyriboletus* species were used as outgroup based on the phylogeny in Vadthanarat et al. (2019). Statistical support of clades was obtained with 1,000 rapid BS replicates. For BI, the best-fit model of substitution among those implementable in MrBayes was estimated separately for each character set using jModeltest (Darriba et al., 2012) on the CIPRES portal, based on the Bayesian information criterion. The selected models were HKY + I + G for *atp*6, SYM + I + G for *tef*1 exons, and K80 + I + G for *rpb*2 exons and intron partition. The Bayesian analysis was performed with MrBayes 3.2.6 software for Windows (Ronquist et al., 2012).

Then, phylogenetic inference of a combined, partitioned dataset was performed, using ML under the same model mentioned above. The phylogenetic tree was inferred from a single analysis with four character sets: *atp*6, *tef*1 exons, *rpb*2 exons and the three introns of *tef*1 + the intron of *rpb*2. The same combined dataset was also analyzed by BI, based on the best-fit model mentioned previously. The two runs of five chains were run for 1,000,000 generations and sampled every 200 generations. At the end of the run, the average deviation of split frequencies was 0.008306, and the potential scale reduction factor values of all parameters were close to 1. The burn-in phase (25%) was estimated by checking the stationarity in the plot generated by the sump command. A total of 7,396 stationary trees were used to reconstruct a 50% majority rule consensus tree and calculate the Bayesian PPs.

## Results

### Phylogenetic Analyses

Fifty-four sequences from *Sutorius* collections corresponding to 11 phylogenetic species were newly generated and deposited in GenBank. The combined alignment of the three loci studied contained 190 sequences (32 for *atp*6, 80 for *tef*1, 78 for *rpb*2) from 80 specimens (Table 1) and was 2,681 characters long (gaps included) (TreeBase no. 27286).

**TABLE 1:** List of collections used for DNA analyses of the genus *Sutorius*, with origin, GenBank accession numbers, and reference(s).

Species	Voucher	Origin	*atp*6	*tef*1	*rpb*2	Reference(s)
*Butyriboletus appendiculatus*	VDKO0193b	Belgium	MG212537	MG212582	MG212624	Vadthanarat et al. (2018)
*Butyriboletus* cf. *roseoflavus*	OR0230	China	KT823974	KT824040	KT824007	Raspé et al. (2016)
*Butyriboletus pseudoregius*	VDKO0925	Belgium	MG212538	MG212583	MG212625	Vadthanarat et al. (2018)
*Caloboletus firmus*	BOS-372	Belize	—	MK721080	MK766288	Kuo and Ortiz-Santana (2020)
*Caloboletus inedulis*	MICH:KUO-07031403	United States	—	MK721081	MK766289	Kuo and Ortiz-Santana (2020)
*Caloboletus radicans*	VDKO1187	Belgium	MG212540	MG212584	MG212626	Vadthanarat et al. (2018)
*Caloboletus yunnanensis*	HKAS69214	China	—	KJ184568	KT990396	Zhao et al. (2014); Wu et al. (2016)
*Crocinoboletus* cf. *laetissimus*	OR0576	Thailand	KT823975	KT824041	KT824008	Raspé et al. (2016)
*Crocinoboletus rufoaureus*	HKAS53424	China	—	KF112206	KF112710	Wu et al. (2014)
*Neoboletus brunneissimus*	HKAS52660	China	—	KF112143	KF112650	Wu et al. (2014)
*Neoboletus ferrugineus*	HKAS77617	China	—	KT990788	KT990430	Wu et al. (2016)
*Neoboletus ferrugineus*	HKAS77718	China	—	KT990789	KT990431	Wu et al. (2016)
*Neoboletus flavidus*	HKAS58724	China	—	KU974137	KU974145	Wu et al. (2016)
*Neoboletus flavidus*	HKAS59443	China	—	KU974136	KU974144	Wu et al. (2016)
*Neoboletus hainanensis*	HKAS63515	China	—	KT990808	KT990449	Wu et al. (2016)
*Neoboletus hainanensis*	HKAS74880	China	—	KT990790	KT990432	Wu et al. (2016)
*Neoboletus hainanensis*	HKAS90209	China	—	KT990809	KT990450	Wu et al. (2016)
*Neoboletus hainanensis*	HKAS59469	China	—	KF112175	KF112669	Wu et al. (2016)
*Neoboletus junquilleus*	AF2922	France	MG212552	MG212596	MG212638	Vadthanarat et al. (2018)
*Neoboletus magnificus*	HKAS54096	China	—	KF112149	KF112654	Wu et al. (2014)
*Neoboletus magnificus*	HKAS74939	China	—	KF112148	KF112653	Wu et al. (2014)
*Neoboletus obscureumbrinus*	HKAS63498	China	—	KT990791	KT990433	Wu et al. (2016)
*Neoboletus obscureumbrinus*	HKAS77774	China	—	KT990792	KT990434	Wu et al. (2016)
*Neoboletus obscureumbrinus*	HKAS89027	China	—	KT990794	KT990436	Wu et al. (2016)
*Neoboletus rubriporus*	HKAS57512	China	—	KF112151	KF112656	Wu et al. (2014)
*Neoboletus rubriporus*	HKAS83026	China	—	KT990795	KT990437	Wu et al. (2016)
*Neoboletus rubriporus*	HKAS89174	China	—	KT990796	KT990438	Wu et al. (2016)
*Neoboletus sanguineoides*	HKAS57766	China	—	KT990799	KT990440	Wu et al. (2016)
*Neoboletus sanguineoides*	HKAS74733	China	—	KT990800	KT990441	Wu et al. (2016)
*Neoboletus sanguineoides*	HKAS55440	China	—	KF112145	KF112652	Wu et al. (2014)
*Neoboletus sanguineus*	HKAS80823	China	—	KT990802	KT990442	Wu et al. (2016)
*Neoboletus sanguineus*	HKAS68587	China	—	KF112150	KF112657	Wu et al. (2014)
*Neoboletus sanguineus*	HKAS90211	China	—	KT990804	KT990444	Wu et al. (2016)
*Neoboletus* sp.	HKAS76660	China	—	KF112180	KF112731	Wu et al. (2014)
*Neoboletus* sp.	OR0128	Thailand	MH614686	MH614734	MH614781	Vadthanarat et al. (2019)
*Neoboletus* sp.	HKAS76851	China	—	KF112144	KF112651	Wu et al. (2014)
*Neoboletus tomentulosus*	HKAS77656	China	—	KT990806	KT990446	Wu et al. (2016)
*Neoboletus tomentulosus*	HKAS53369	China	—	KF112154	KF112659	Wu et al. (2014)
*Neoboletus tomentulosus*	HKAS77614	China	—	KT990805	KT990445	Wu et al. (2016)
*Neoboletus venenatus*	HKAS63535	China	—	KT990807	KT990448	Wu et al. (2016)
*Pulveroboletus* aff. *ravenelii*	ADK4360	Togo	KT823957	KT824023	KT823990	Raspé et al. (2016)
*Pulveroboletus* aff. *ravenelii*	ADK4650	Togo	KT823959	KT824025	KT823992	Raspé et al. (2016)
*Pulveroboletus* aff. *ravenelii*	HKAS53351	China	—	KF112261	KF112712	Wu et al. (2014)
*Pulveroboletus fragrans*	OR0673	Thailand	KT823977	KT824043	KT824010	Raspé et al. (2016)
*Pulveroboletus ravenelii*	REH2565	United States	KU665635	KU665636	KU665637	Raspé et al. (2016)
*Pulveroboletus* sp.	HKAS57665	China	—	KF112264	KF112715	Wu et al. (2014)
*Pulveroboletus* sp.	HKAS74933	China	—	KF112262	KF112713	Wu et al. (2014)
*Rubroboletus legaliae*	VDKO0936	Belgium	KT823985	KT824051	KT824018	Raspé et al. (2016)
*Rubroboletus rhodoxanthus*	HKAS84879	China	—	KT990831	KT990468	Wu et al. (2016)
*Rubroboletus satanas*	VDKO0968	Belgium	KT823986	KT824052	KT824019	Raspé et al. (2016)
*Suillellus luridus*	VDKO0241b	Belgium	KT823981	KT824047	KT824014	Raspé et al. (2016)
*Suillellus subamygdalinus*	HKAS53641	China	—	KT990841	KT990478	Wu et al. (2016)
*Sutorius* aff. *eximius*	HKAS50420	China	—	KT990750	KT990387	Wu et al. (2016)
*Sutorius* aff. *eximius*	HKAS52672	China	—	KF112207	KF112802	Wu et al. (2014)
*Sutorius* aff. *eximius*	HKAS56291	China	—	KF112208	KF112803	Wu et al. (2014)
*Sutorius* aff. *eximius*	HKAS59657	China	—	KT990887	KT990505	Wu et al. (2016)
*Sutorius* aff. *eximius*	TWO-986	Costa Rica	—	MK721180	MK766383	Kuo and Ortiz-Santana (2020)
*Sutorius australiensis*	REH9441	Australia	MG212567	JQ327032*	MG212652	Vadthanarat et al. (2018); *Halling et al. (2012)
*Sutorius australiensis*	REH9485	Australia	—	MK721165	MK766367	Kuo and Ortiz-Santana (2020)
*Sutorius australiensis*	REH10021	Australia	—	MK721166	MK766368	Kuo and Ortiz-Santana (2020)
*Sutorius eximius*	REH9400	United States	MG212568	JQ327029*	MG212653	Vadthanarat et al. (2018); *Halling et al. (2012)
*Sutorius eximius*	REH10038	United States	—	MK721167	MK766369	Kuo and Ortiz-Santana (2020)
*Sutorius maculatoides*	OR0758	Thailand	**MN067462**	**MN067481**	**MN067498**	This study
*Sutorius maculatoides*	WAT25793	Malaysia	**MN067463**	**MN067482**	—	This study
*Sutorius maculatoides*	OR0626	Thailand	**MN067461**	**MN067480**	—	This study
*Sutorius mucosus*	OR0851 (type)	Thailand	**MN067464**	**MN067483**	**MN067499**	This study
*Sutorius pachypus*	OR0411 (type)	Thailand	**MN067465**	**MN067484**	**MN067500**	This study
*Sutorius pachypus*	SV0098	Thailand	**MN067466**	**MN067485**	**MN067501**	This study
*Sutorius pachypus*	TWO1171	Thailand	**MW194871**	—	—	This study
*Sutorius pseudotylopilus*	OR0378B (type)	Thailand	MH614692	MH614740	MH614787	Vadthanarat et al. (2019)
*Sutorius pseudotylopilus*	SV0401	Thailand	**MN067467**	**MN067486**	**MN067502**	This study
*Sutorius pseudotylopilus*	SV0415	Thailand	**MN067468**	**MN067487**	**MN067503**	This study
*Sutorius rubinus*	OR0379	Thailand	MH614693	MH614741	MH614788	Vadthanarat et al. (2019)
*Sutorius rubinus*	OR0403 (type)	Thailand	**MN067469**	**MN067488**	**MN067504**	This study
*Sutorius rubinus*	OR0409	Thailand	**MN067470**	**MN067489**	**MN067505**	This study
*Sutorius rubinus*	OR1255	Thailand	**MN067471**	**MN067490**	**MN067506**	This study
*Sutorius* sp.	ADK2396	Zimbabwe	**MN067477**	**MN067495**	**MN067511**	This study
*Sutorius* sp.	ADK4369	Togo	**MN067478**	**MN067496**	**MN067512**	This study
*Sutorius* sp.	JD669	Burundi	**MN067479**	**MN067497**	**MN067513**	This study
*Sutorius subrufus*	FHMU2101	China	—	MH879730	MH879747	Chai et al., 2019
*Sutorius subrufus*	FHMU2006	China	—	MH879729	MH879746	Chai et al., 2019
*Sutorius ubonensis*	SV0032 (type)	Thailand	**MN067472**	**MN067491**	**MN067507**	This study
*Sutorius ubonensis*	SV0203	Thailand	**MN067473**	**MN067492**	**MN067508**	This study
*Sutorius ubonensis*	SV0353	Thailand	**MN067474**	**MN067493**	**MN067509**	This study
*Sutorius obscuripellis*	OR0949 (type)	Thailand	**MN067475**	**MN067494**	**MN067510**	This study
*Sutorius vellingae*	ECV3603 (type)	Thailand	**MN067476**	JQ327033	—	This study; Halling et al. (2012)

*Newly generated sequences are shown in bold font.*

The single-gene trees were very similar in topology (Supplementary Figures 1–4), except for one supported conflict. In the *tef*1 exons tree (Supplementary Figure 2), *Sutorius pachypus* formed a clade sister to *S*. *australiensis* with high support (BS = 75%, PP = 0.92). This is in conflict with the topology of the tree based on the introns character set (Supplementary Figure 4), in which *S*. *pachypus* formed a clade sister to *Sutorius rubinus* (BS = 87%, PP = 0.97). Partitioned analyses of a dataset combining *atp*6, *tef*1 exons, and *rpb*2 exons sequences were also performed (Supplementary Figure 5). Its tree topology showed no conflict to the single partitions of *atp*6, *tef*1 exons, and *rpb*2 exons trees. Consequently, the combination of four-partition analyses was used to infer the relationship between species in this study.

**FIGURE 1:**
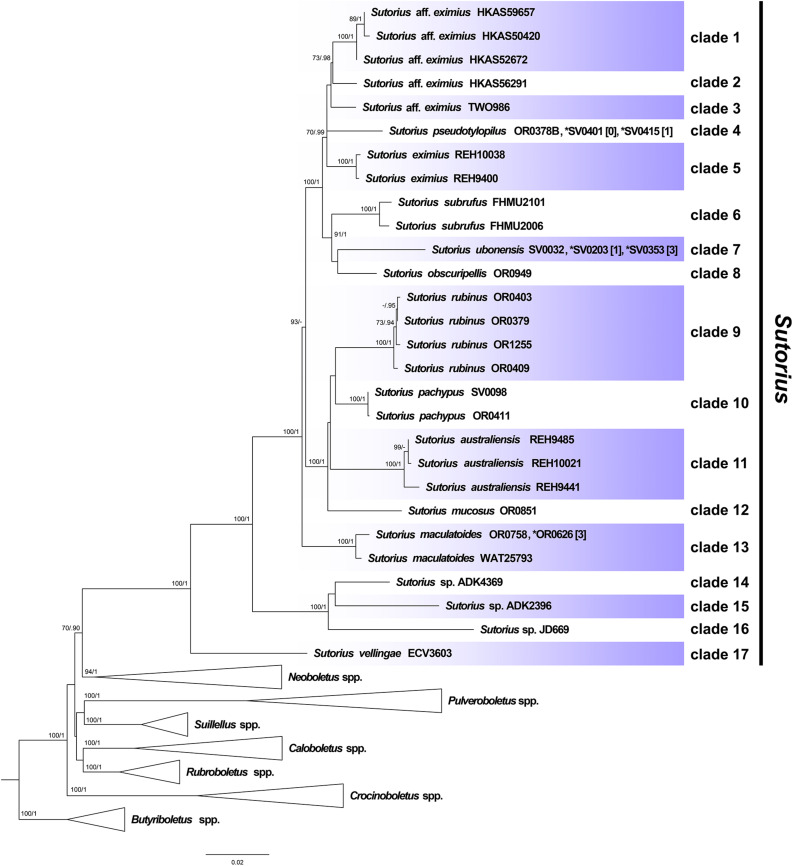
Phylogenetic tree inferred from the three-gene dataset (*atp*6, *rpb*2 and *tef*1), of *Sutorius* species and selected Boletaceae in *Pulveroboletus* group, using Maximum Likelihood and Bayesian Inference methods (ML bipartition tree is presented). The three *Butyriboletus* species were used as outgroup. All generic clades, excluding *Sutorius*, that were highly supported were collapsed. Seventeen species-level clades within *Sutorius* are indicated with label. Bootstrap support values (BS ≥ 70%) and posterior probabilities (PP ≥ 0.90) are shown above the supported branches. The star (*) indicates additional collections with exactly identical sequences or sequences differing only by heteromorphisms in *tef*1 (with the number of heteromorphisms mentioned in square brackets [ ]).

Maximum likelihood and BI trees of the combined three-gene dataset showed similar topologies without any supported conflict (Bootstrap support values, BS ≥ 70% and PP ≥ 0.90; Figure 1). The phylogram of RAxML bipartitions (Figure 1) indicated that all selected Boletaceae formed eight highly supported generic clades (BS = 100% and PP = 1 for *Sutorius*, *Pulveroboletus*, *Suillellus*, *Rubroboletus*, *Crocinoboletus*, and *Butyriboletus*; BS = 94% and PP = 1 for the *Neoboletus* clade). All *Sutorius* exemplars clustered together in a strongly supported clade, sister to *Neoboletus* (BS = 70% and PP = 0.90). In the *Sutorius* clade, 17 terminal, species-level clades were resolved with high support, including the three previously described species clades (clade 5 = *S*. *eximius*, clade 6 = *S*. *subrufus*, and clade 11 = *S*. *australiensis*); 7 clades of new, undescribed species originating from Thailand (clades 4, 7, 8, 9, 10, 12, and 17), with one clade (clade 13) containing *T. maculatoides* (WAT25793); and two Thai collections (OR0626 and OR0758). Also, six clades of unnamed species were inferred (clades 1 and 2 from China; clade 3 from Costa Rica; clades 14, 15, and 16 from Togo, Zimbabwe, and Burundi, respectively).

### Taxonomy

***Sutorius maculatoides*** (E. Horak) Vadthanarat, Raspé and Lumyong **comb. nov.**

MycoBank: MB838055

Figures 2, 3

**FIGURE 2:**
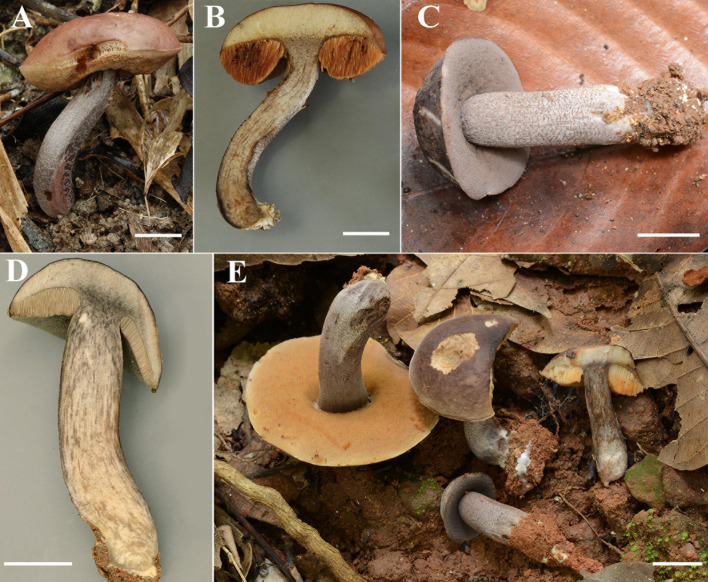
**(A–E)** Fresh basidiomata of *Sutorius maculatoides* (**A,B**: OR0626; **C,D**: OR0754; **E**: OR758). Scale bars: **(A–E)** = 1 cm.

**FIGURE 3:**
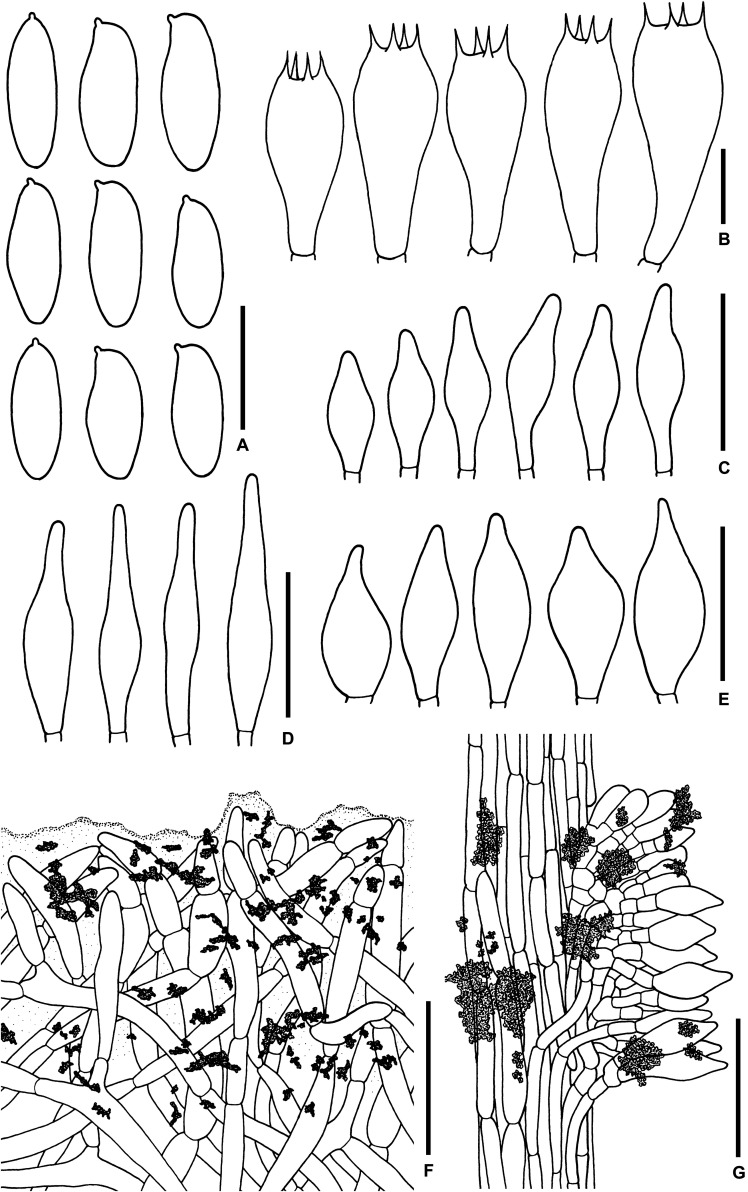
Microscopic features of *Sutorius maculatoides*. **(A)** Basidiospores. **(B)** Basidia. **(C)** Cheilocystidia. **(D)** Pleurocystidia. **(E)** Caulocystidia. **(F)** Pileipellis. **(G)** Stipitipellis. All drawings were made from OR0758. Scale bars: **(A,B)** = 10 μm; **(C–E)** = 25 μm; **(F,G)** = 50 μm.

Basionym: *B. maculatus* Corner, *Boletus* in Malaysia: 160, 1972, non-Raddi, Mem. Mat. Fis. Soc. Ital. Sci. Modena, Pt. Mem. Fis. 13: 352. 1807.

Synonymy: *T. maculatoides* E. Horak, Malayan Forest Records 51: 98, 2011.

*Description* (based on Thai specimens): *Basidiomata* small- to medium-sized. *Pileus* (1.8) 2.4 to 4.6 cm in diameter, at first hemispherical with straight margin becoming convex with deflexed margin; *surface* subrugulose, dull, minutely tomentose, at first dark lilac (10–11F3–6) becoming purplish to reddish brown to brown (8F6, 7E5) in age, gradually paler to the margin. *Pileus context* 4–5 mm thick halfway to the margin, firm, yellowish off-white (4A2), near the center grayish to purplish white (14–15C–E2) at first, then yellowish white (4A2) in age, with scattered small violet–brown (11F4–6) encrustations. *Stipe* central, terete to slightly compressed, cylindrical, slightly wider at the base, 2.4–4.5 × 0.6–1.2 cm; *surface* finely scabrous with groups of brown to dark brown (7–8F4–6) granulose squamules on purplish gray (15C2, 15D3) background; *basal tomentum* little developed, white. *Stipe context* solid, pale grayish orange (5B2), virgated with grayish purple (15–16C3), with scattered small violet–brown (11F4–6) encrustations. *Hymenophore* tubulose, subventricose to adnexed to broadly adnexed, depressed around stipe; *tubes* easily separable, at first grayish brown (7D3) then brownish orange (6C6) with age, 8–9 mm long halfway to the margin; *pores* 0.2–0.4(0.7) mm wide at midradius, regular roundish; at first lilac-gray (15–16E3 to 15C2) slightly paler near the margin, becoming pale reddish brown (9D–E4) with age. *Odor* fungoid. *Taste* mild. *Spore print* reddish brown (8E/F5).

*Macrochemical reactions*: KOH: pale greenish yellow on pileus, pileus context, stipe and stipe context; pale brownish orange on hymenophore. NH_4_OH: pale greenish yellow on pileus, on pileus context bluish white (21A2) when young or negative in mature basidiomes; greenish yellow on stipe; greenish yellow then purplish on stipe context.

*Basidiospores* [165/3/3] (7.8–) 8.3–10.9–13.2 (–14.1) × (3–) 3.3–4–4.7 (–5.6) μm, *Q* = (2.02–) 2.24–2.69–3.18 (–3.41), narrowly ellipsoid to subcylindrical with slight suprahilar depression, thin-walled, smooth, brownish to yellowish hyaline in water, yellowish hyaline in KOH or NH_4_OH, inamyloid. *Basidia* 4-spored (23–) 23–26–31 (–32) × (10–) 10–11–13 (–14) μm, with sterigmata up to 4 μm long, clavate, hyaline, inamyloid. *Cheilocystidia* (19–) 19–22–27 (–27) × (6–) 6–8–9 (–9) μm, frequent, fusiform, thin-walled, hyaline. *Pleurocystidia* (32–) 32–38–45 (–45) × (6–) 6–8–10 (–10) μm, infrequent, narrowly fusiform, thin-walled, hyaline. *Hymenophoral trama* divergent becoming boletoid in age, 21–52 μm wide, with regular mediostratum 9–22 μm wide. *Pileipellis* a sub-ixotrichoderm to intricate trichoderm, 135–212 μm thick, terminal cells (12–55 × 5–11 μm) fusiform with slightly tapering apex, pale yellowish brown in water, mostly hyaline to pale yellowish brown in KOH or NH_4_OH, with scattered loose crystals. *Pileus context* made of strongly interwoven, hyaline, thin-walled hyphae, 8–13 μm wide, with scattered loose crystals. *Stipitipellis* a disrupted hymeniderm, 100–120 μm thick, composed of parallel hyphae, with terminal cell 11–33 × 7–10 μm of thin-walled, hyaline hyphae, giving rise to clusters of caulocystidia and basidiole-like cells, with scattered loose crystals. *Caulocystidia* (18–) 18–26–37 (–38) × (8–) 8–11–15 (–15) μm, frequent, fusiform to broadly fusiform with subacute apex, thin-walled, hyaline. *Stipe context* composed of parallel, 4- to 12-μm-wide hyphae, with scattered loose crystals. *Clamp connections* not seen in any tissue.

*Microscopic description* (based on WAT25793): *Basidiospores* (11.4–) 11.5–12.1–12.7 (–13.5) × (4.1–) 4.2–4.5–4.8 (–4.9) μm, *Q* = (2.42–) 2.42–2.68–2.94 (–3.05), *N* = 72, narrowly ellipsoid to subcylindrical with slight suprahilar depression, thin-walled, smooth, brownish to yellowish hyaline in water, yellowish hyaline in KOH and NH_4_OH, inamyloid. *Basidia* 4-spored (21–) 21–24–28 (–29) × (9–) 10–11–12 (–12) μm, with sterigmata up to 3 μm long, clavate, yellowish hyaline to yellowish-brown in KOH, inamyloid. *Cheilocystidia* (15–) 16–21–24 (–24) × (6–) 6–7–8 (–8) μm, frequent, fusiform, thin-walled hyaline. *Pleurocystidia* (31–) 31–36–43 (–43) × (5–) 5–6–7 (–7) μm, quite rare, narrowly fusiform, thin-walled, hyaline. *Pileipellis* a sub-ixotrichoderm to intricate trichoderm, 67- to 132-μm-thick, thin-walled hyphae, with fusiform terminal cells (11–53 × 6–11 μm) with slightly tapering apex. *Pileus context* made of moderately interwoven hyaline hyphae, 6–13 μm wide. *Stipitipellis* a disrupted hymeniderm, 65–85 μm thick, composed of parallel hyphae, with rounded terminal cell 14–40 × 5–10 μm of thin-walled, hyaline hyphae; at places the terminal cells grouped with caulocystidia into clusters, with scattered loose crystals. *Stipe context* composed of parallel 4- to 14-μm-wide hyaline, thin-walled hyphae. *Caulocystidia* (20–) 20–28–37 (–37) × (7–) 8–11–15 (–15) μm, frequent, fusiform to broadly fusiform with subacute apex, thin-walled, hyaline. *Clamp connections* not seen in any tissue.

*Habitat*: Solitary to gregarious on soil, in hill forest dominated by *Dipterocarpus obtusifolius*, *Dipterocarpus tuberculatus*, *Shorea obtusa*, and *Shorea siamensis*.

*Distribution*: Malaysia, Singapore, and Thailand.

*Collections examined*: **MALAYSIA:** Forest Research Institute Kepong, MNS foray along rovers’ trail, 27 February 1994, Roy Watling, WAT25793 (E). **THAILAND:** Chiang Mai Province: Doi Suthep-Pui National Park, 18°48′47″N–98°56′11″E, elev. 540 m, May 18, 2015, Olivier Raspé, OR0626 (BKF, CMUB); –ibid., 18°47′37″N–98°55′41″E, elev. 770 m, May 24, 2015, Olivier Raspé and Santhiti Vadthanarat, OR0754 (BKF, CMUB); –ibid., 18°47′38″N–98°55′42″E, elev. 770 m, May 26, 2015, Olivier Raspé and Santhiti Vadthanarat, OR0758 (BKF, CMUB); Mae On District, Ban Huay Kaew community forest, 18°52′7.15″N–99°17′38.92″E. 750 m, July 17, 2017, Olivier Raspé, OR1414 (CMUB, BR).

Notes: Corner (1972) described *B. maculatus* from Malaysia. However, it was a later non-homotypic homonym of *B. maculatus* Raddi described in 1807. Consequently, Horak (2011) gave the new epithet *maculatoides*. He also transferred the species to *Tylopilus*. Here, we present phylogenetic evidence supporting placement in *Sutorius*. In our phylogenetic inference (Figure 1), all *S*. *maculatoides* collections clustered together in a well-supported terminal clade (clade 13), within the *Sutorius* clade. The macromorphological and micromorphological characters of *S*. *maculatoides* also support its position in *Sutorius*. Therefore, the new combination *S. maculatoides* is proposed for *T. maculatoides*. In our phylogeny, among the *S*. *maculatoides* specimens, the Thai collections (OR0626 and OR0758) were molecularly slightly divergent from the Malaysian specimen (WAT25793). Being the amount of divergence is lower than the one observed between sister species, we consider those differences in the range of intraspecific genetic variability and compatible with conspecific, geographically distant populations.

*Sutorius maculatoides* is characterized by the following characteristics: dark lilac pileus with lilac gray pores at first, with age becoming reddish brown and reddish pale brown, respectively; presence of cheilocystidia and infrequent pleurocystidia; pileipellis a sub-ixotrichoderm composed of moderately interwoven hyphae, with fusiform terminal cells that slightly taper at the apex.

In this study, *S. maculatoides* is redescribed based on specimens from Thailand (OR0626 and OR0758) and Malaysia (WAT25793), which were compared to the description of *T. maculatoides* E. Horak (Horak, 2011). Microcharacteristics of Watling’s specimen were similar to Horak’s description including size and shape of basidiospores, basidia, and pileipellis. However, the cheilocystidia in Watling’s specimen (15–24 × 6–8 μm) are shorter than in Horak’s description (35–45 × 6–11 μm), but Horak did not separately describe the length of cheilocystidia and pleurocystidia. He also did not mention the frequency of both types of cystidia. By comparison, microscopic characters between Watling’s specimen and the Thai specimens were almost identical. However, the minimum value of the length of basidiospores in Watling’s specimen (11.4–13.5 × 4.1–4.9 μm) is higher than for the Thai specimens (7.8–14.1 × 3–5.6 μm). The lower minimum value of length of basidiospores in Thai specimens could be explained by small quantitative differences between geographically distant populations or the less mature stage of some basidiomata from Thai collections. Moreover, according to our observations on *Sutorius*, this genus usually shows high within-species variation in spore size, which was also noted originally by Halling et al. (2012).

***Sutorius mucosus*** Vadthanarat, Raspé and Lumyong **sp. nov.**

MycoBank: MB838057

Figures 4A–C, 5

**FIGURE 4:**
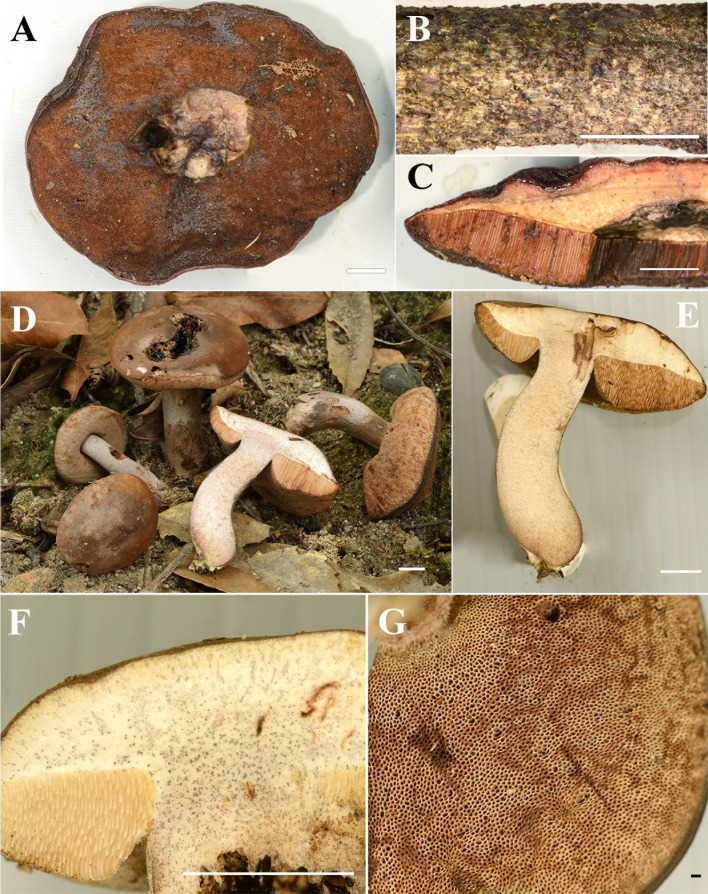
**(A–C)** Fresh basidiomata of *Sutorius mucosus* specimen voucher OR0851 holotype (**A**: pores surface, **B**: stipe surface, **C**: slimy pileus and context with tubes). **(D–G)** Fresh basidiomata of *Sutorius obscuripellis* OR0949 holotype. Scale bars: **(A–F)** = 1 cm, **(G)** = 1 mm.

**FIGURE 5:**
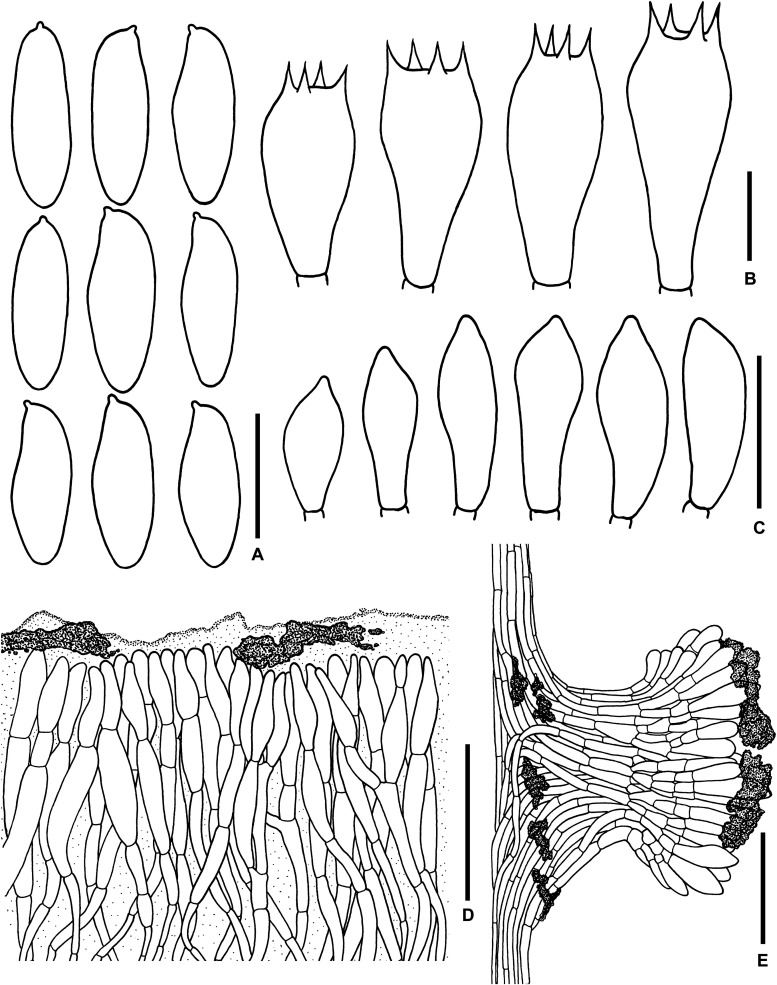
Microscopic features of *Sutorius mucosus*. **(A)** Basidiospores, **(B)** Basidia, **(C)** Caulocystidia, **(D)** Pileipellis, **(E)** Stipitipellis. All drawings were made from OR0581. Scale bars: **(A,B)** = 10 μm; **(C)** = 25 μm; **(D,E)** = 50 μm.

*Typification*: **THAILAND:** Chiang Mai Province: Mae Taeng District, Pha Deng village, 19°6′31″N–98°44′30″E, elev. 1,050 m, July 29, 2014, Olivier Raspé and Anan Thawthong, OR0851 (MFLU: holotype; BR: isotype).

*Etymology*: from Latin mucosus, means mucus, referring to a strongly gelatinized pileipellis.

*Description*: *Basidiomata* medium-sized. *Pileus* 9 cm in diameter, planoconvex with straight margin, margin slightly exceeding (1 mm); *surface* subrugulose, dull, waxy to subviscid, almost smooth to minutely tomentose under the lens, reddish brown (8E/F8). *Pileus context* 8–10 mm halfway to the margin, soft, dull yellowish white (4A2), with scattered small groups of reddish brown (9F5–7) encrustations, slightly reddening (5A2–3) when cut. *Stipe* central, terete, cylindrical, × 1.2 cm; *surface* subscabrous, with granulose squamules, purplish brown to dark brown (10–11F4–7), uplifted by pale brown (5B/C3–5) hyphae; *stipe context* solid, slightly turgid, beige (4B/C3). *Hymenophore* tubulose, ventricose; *tubes* easily separable, pale orange (5A2–3), 7–9 mm long halfway to the margin; *pores* 0.2–0.3(0.5) mm wide at midradius, roundish to angular near the stipe, pore topography irregular, brown to brownish gray (7B4–5 to 8D/E4). *Odor* and *taste* not observed. *Spore print* reddish brown (8E/F8).

*Macrochemical reactions*: KOH: yellowish on pileus context.

*Basidiospores* (10.4–) 10.8–13.8–15 (–15.4) × (4.3–) 4.6–5–5.5 (–5.6) μm, *Q* = (2.24–) 2.47–2.76–3.04 (–3.06), *N* = 55, narrowly ellipsoid to subcylindrical with slight suprahilar depression, thin-walled, smooth, brownish to yellowish hyaline in water, yellowish hyaline in KOH or NH_4_OH, inamyloid. *Basidia* 4-spored (23–) 23–26–30 (–30) × (10–) 10–11–13 (–13) μm, with sterigmata up to 4 μm long, clavate, hyaline, inamyloid. *Cheilocystidia* and *Pleurocystidia* not seen. *H. trama* slightly divergent to boletoid 17–42 μm wide, with subregular mediostratum 7–12 μm wide. *Pileipellis* an ixotrichoderm, 75–125 μm thick, made of moderately interwoven, gelatinized, hyaline hyphae, with terminal cells 11–37 × 7–11 μm, fusiform to utriform with rounded to tapping apex, pale yellowish to brownish hyaline in water, mostly hyaline to yellowish hyaline in KOH or NH_4_OH, with scattered loose crystals. *Pileus context* composed of strongly gelatinized hyaline hyphae 8–16 μm wide, at places with scattered loose crystals. *Stipitipellis* a disrupted hymeniderm 200–450 (600) μm thick composed of parallel hyphae, with terminal cell 15–29 × 6–9 μm of thin-walled, hyaline hyphae, giving rise to clusters of basidiole-like cells and caulocystidia, with scattered loose crystals. *Caulocystidia* (19–) 19–27–36 (–36) × (8–) 8–11–15 (–15) μm, not frequent, fusiform to broadly fusiform, thin-walled, hyaline. *Stipe context* composed of parallel, 8- to 12-μm-wide gelatinized hyphae, somewhere covered with some encrustations on the wall, with scattered loose crystals. *Clamp connections* not seen.

*Habitat*: Solitary on soil in evergreen hill forest dominated by *Castanopsis* spp., *Lithocarpus* spp. mixed with *D. obtusifolius*, *Dipterocarpus costatus*, *Sh. obtusa*, and *Sh*. *siamensis*.

*Distribution*: Chiang Mai Province, Northern Thailand.

*Notes*: *S. mucosus* description is based on a single collection (OR0851); however, the strongly gelatinized pileipellis is a strikingly unique character, which, together with the phylogenetic evidence, which resolved this specimen as an isolated branch, within the *Sutorius* lineage, supports our interpretation of OR0851 as a new, distinct species. Other important characters that allow differentiating *S. mucosus* are the fusiform to utriform pileipellis terminal cells with rounded to subacute apex and the lack of cheilocystidia and pleurocystidia.

Phylogenetically, *S. mucosus* is resolved as an isolated branch, within a clade comprising three other distinct *Sutorius* species, including *S*. *rubinus* (clade 9), *S. pachypus* (clade 10), and *S*. *australiensis* (clade 11). However, they are morphologically different, and none of them has a strong gelatinized pileipellis, which is the most striking diagnostic character of *S. mucosus*. The other differences are as follows: *S. pachypus* has, on average, a wider stipe (2.5–4 cm wide) and paler stipe surface, presence of cheilocystidia and pleurocystidia, and palisadodermal pileipellis composed of subcylindrical terminal cells with rounded to subacute apex. *S. australiensis* has a trichoderm pileipellis with elongated to cylindrical hyphae with obtuse apex terminal cells. *S. rubinus* also has a trichoderm pileipellis but with different shapes of terminal cells, which are fusiform to broadly fusiform with acuminate to subacute or acute apex, and presence of cheilocystidia.

***Sutorius obscuripellis*** Vadthanarat, Raspé and Lumyong **sp. nov.**

MycoBank: MB838059

Figures 4D–G, 6

**FIGURE 6:**
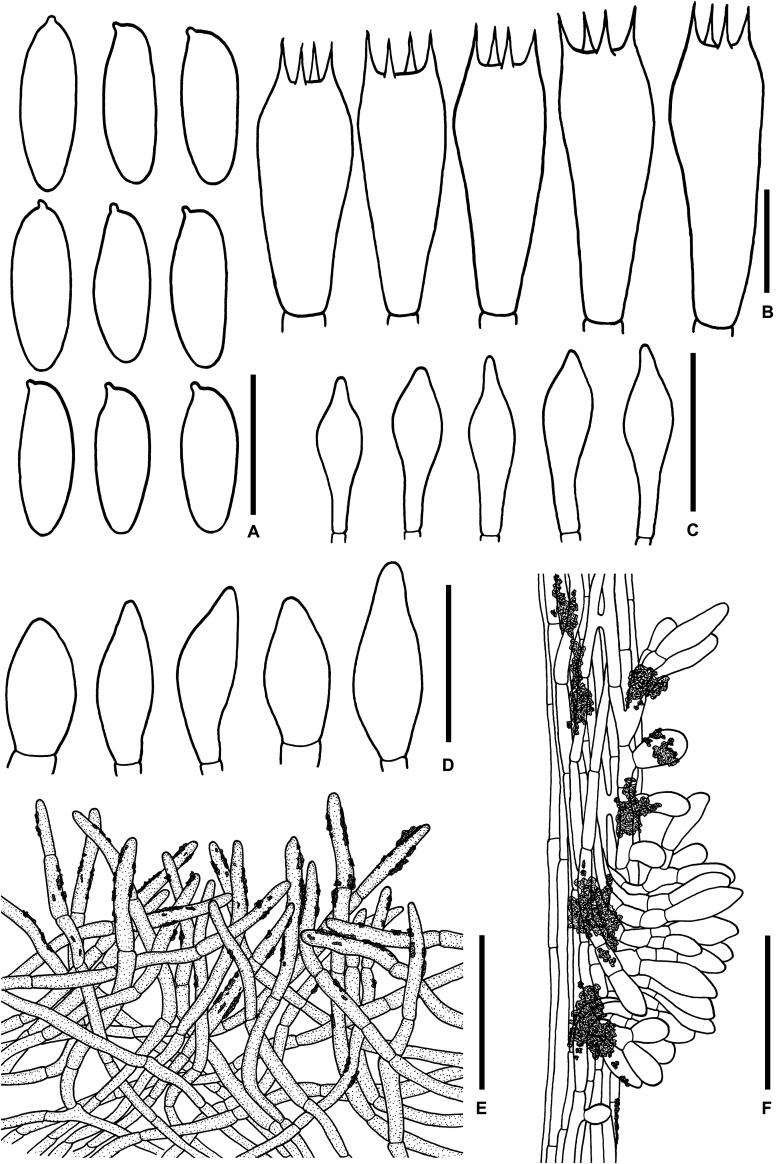
Microscopic features of *Sutorius obscuripellis*. **(A)** Basidiospores. **(B)** Basidia. **(C)** Cheilocystidia. **(D)** Caulocystidia. **(E)** Pileipellis. **(F)** Stipitipellis. All drawings were made from OR0949. Scale bars: **(A,B)** = 10 μm; **(C,D)** = 25 μm; **(E,F)** = 50 μm.

*Typification*: **THAILAND:** Chiang Mai Province: Mae On District, Pok village, 18°53′11″N–99°22′11″E, elev. 710 m, July 29, 2015, Olivier Raspé, OR0949 (CMUB: holotype, BR: isotype).

*Etymology*: from Latin *obscurus* and *pellis*, referring to the dark pileipellis.

*Description*: *Basidiomata* small- to medium-sized. *Pileus* (3.5) 4.5–6 cm in diameter convex to planoconvex, with straight margin; *surface* even, minutely and densely matted tomentose, dull, violet–brown to dark brown (10F5–6 to 9E4), with patchy color variation, sometimes gradually paler to margin. *Pileus context* 6–9 mm thick halfway to the margin, yellowish white (4A2), more brownish near the pellis, slowly turning pale red–brown when cut, with scattered small groups of reddish brown to dark brown (9E4–5, 9F5–6) encrustations. *Stipe* clavate to cylindrical (3.5) 3.8–5.5 × 0.8–1.7 cm; *surface* dull, even to finely scabrous, violet-gray (18D2, 16E2) with violet–brown (10E43–5) granulose squamules; *basal tomentum* off-white to yellowish white. *Stipe context* solid, fleshy fibrous, light purplish brown (12B2), more purplish at the base, with scattered small groups of reddish brown to dark brown (9E4–5, 9F5–6) encrustations. *Hymenophore* tubulose, adnexed to adnate, depressed around stipe, ventricose; *tubes* orange–gray (6B2), 6–13 mm long, easily separable, turning red–brown on bruising; *pores* 0.2–0.5 mm wide at midradius, roundish, regularly arranged, with subregular pore surface, pale violet-gray (9–10B3), becoming paler with age, slightly reddish brown on bruising, with scattered small groups of reddish brown to dark brown encrustations, composite pores uncommon. *Odor* not recorded. *Taste* slightly bitter at first, then mild. *Spore print* not recorded.

*Macrochemical reactions*: KOH: dull yellow on pileus; yellow or pale yellow on pileus context, stipe, stipe context, and hymenophore. NH_4_OH: yellowish with only slight violet aura on pileus; yellowish on stipe and pileus context; negative on hymenophore and stipe context.

*Basidiospores* (9.6–)9.8–10.8–12.2 (–13.5) × (3.6–) 3.8–4.2–4.6 (–5.1) μm, *Q* = (2.19–) 2.34–2.55–2.83 (–2.97), *N* = 72, narrowly ellipsoid to subcylindrical with slightly suprahilar depression, thin-walled, smooth, brownish to yellowish hyaline in water, yellowish hyaline in KOH and NH_4_OH, inamyloid. *Basidia* 4-spored (23–) 23–26–30 (–30) × (8–) 8–10–11 (–11) μm, with sterigmata up to 5 μm long, clavate, hyaline, inamyloid. *Cheilocystidia* (18–) 19–24–30 (–31) × (6–) 6–7–9 (–9) μm, frequent, fusiform, thin-walled, hyaline. *Pleurocystidia* not seen. *H. trama* divergent to boletoid, 47–91 μm wide; with 12–48 μm wide of regular mediostratum. *Pileipellis* an intricate trichoderm, 100–170 μm thick, made of loosely interwoven terminal cells 17–65 × 3–6 μm, with subacute to rounded apex, brown to dark in water especially the terminal cells, less brown in KOH or NH_4_OH. *Pileus context* composed of moderately interwoven, 6- to 12 (15)-μm-wide hyphae, brownish to yellowish hyaline, with some scattered parietal encrustations and loose crystals. *Stipitipellis* a disrupted hymeniderm 100–125 μm thick, composed of parallel hyphae, anastomosing at places, with terminal cell 10–24 × 6–12 μm of thin-walled hyaline hyphae, giving rise to clusters of caulocystidia and basidiole-like cells, with scattered loose crystals. *Caulocystidia* (19–) 20–26–38 (–38) × (6–) 6–11–20 (–22) μm, broadly fusiform, thin-walled, hyaline. *Stipe context* composed of parallel 5- to 8 (12)-μm-wide hyphae, with scattered loose crystals. *Clamp connections* not seen in any tissue.

*Habitat*: On soil, gregarious to fasciculate in hill forest dominated by *Castanopsis* spp. *Lithocarpus* spp. mixed with some *Dipterocarpus* spp. and regularly subject to fire.

*Distribution*: Chiang Mai Province, Northern Thailand.

*Notes*: Based on a single collection (OR0949), *S. obscuripellis* is characterized by the following combination of characteristics: basidiomata small- to medium-sized; pale violet–gray pores color; lack of pleurocystidia; *Pileipellis* an intricate trichoderm composed of brown to dark hyphae with cylindrical terminal cells with subacute to rounded apex, showing scattered small parietal brownish yellow to reddish pale to dark brown encrustations. Phylogenetically, *S. obscuripellis* formed a clade (clade 8) sister to *Sutorius ubonensis* (clade 7) and *S*. *subrufus* (clade 6). However, the latter two species are morphologically different from *S. obscuripellis* as follows: *S*. *ubonensis* has larger basidiomata, darker pores, and basidiomata especially when young, pleurocystidia, and a slightly gelatinized tomentose pileipellis, and has so far only been found in dry dipterocarp forest in Ubon Ratchathani Province, in northeastern Thailand. *S. subrufus* has larger basidiomata, a stipe surface and context turning more reddish when injured, presence of pleurocystidia, and a trichoderm pileipellis composed of cylindrical hyphae and clavate or subclavate terminal cells with obtuse apex, colorless to yellowish in KOH (Chai et al., 2019), whereas *S. obscuripellis* has the pileipellis composed of brown to dark brown hyphae, colorless to brownish yellow to pale brown in KOH.

***Sutorius pachypus*** Vadthanarat, Raspé and Lumyong **sp. nov.**

MycoBank: MB838060

Figures 7A–E, 8

**FIGURE 7:**
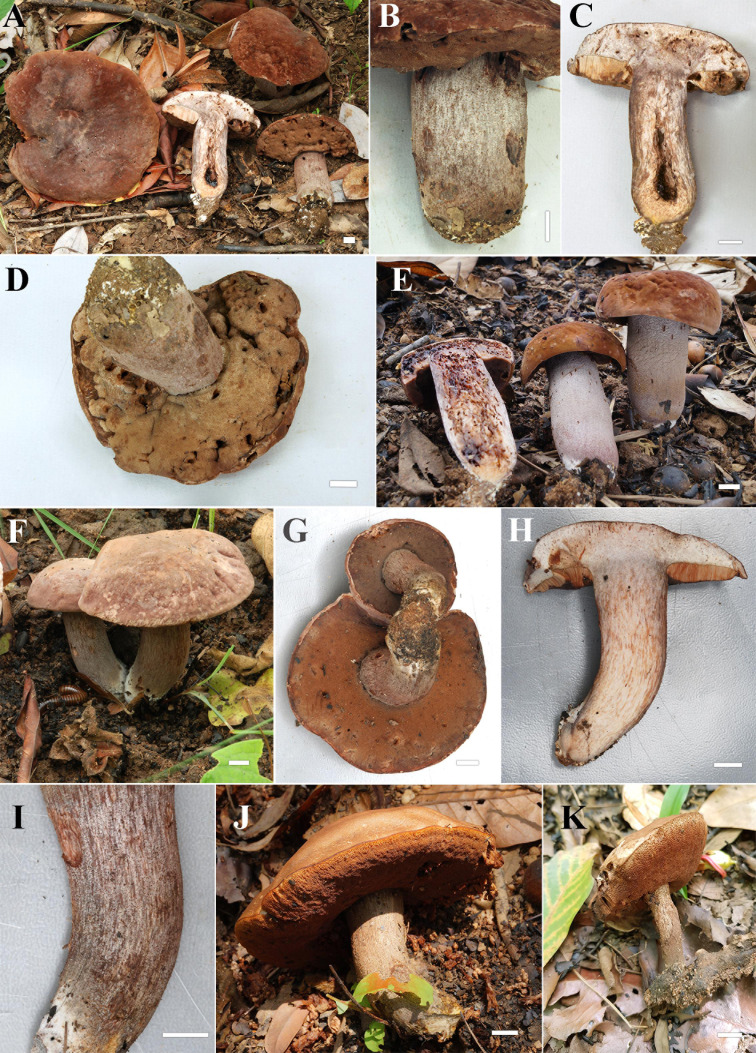
**(A–E)** Fresh basidiomata of *Sutorius pachypus* (**A–D**: OR0411, **E**: SV0098). **(F–K)** Fresh basidiomata of *Sutorius pseudotylopilus* (**F–I**: OR0378B, **J**: SV0401, **K**: SV0415). Scale bars: **(A–K)** = 1 cm.

**FIGURE 8:**
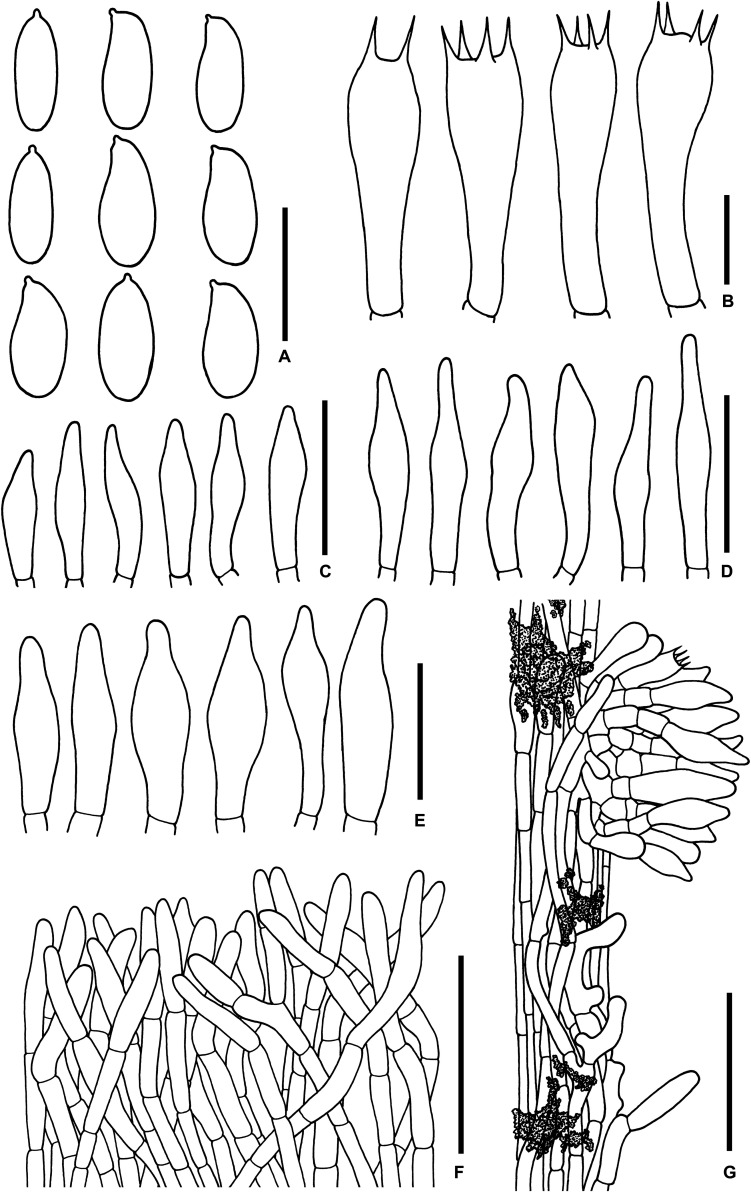
Microscopic features of *Sutorius pachypus*. **(A)** Basidiospores. **(B)** Basidia. **(C)** Cheilocystidia. **(D)** Pleurocystidia. **(E)** Caulocystidia. **(F)** Pileipellis. **(G)** Stipitipellis. All drawings were made from OR0411. Scale bars: **(A,B)** = 10 μm; **(C–E)** = 25 μm; **(F,G)** = 50 μm.

*Typification*: **THAILAND:** Chiang Mai Province: Mae Taeng District, Behind Wat Pah Deng, 19°06′38″N–98°44′32″E, elev. 1,055 m, June 7, 2012, Olivier Raspé and Komsit Wisitrassameewong, OR0411 (MFLU: holotype; BR: isotype).

*Etymology*: from Greek means “thick” referring to the wide stipe.

*Description*: *Basidiomata* medium-sized. *Pileus* 4.5–9 cm in diameter; at first hemispherical then convex to planoconvex with deflexed, slightly exceeding margin (1–2 mm); *surface* subrugulose, dull, minutely tomentose, at first dark reddish brown to dark brown (9F5–6) becoming pale reddish brown (8–9D/E4–7), gradually paler to the margin. *Pileus context* 1.5–1.7 cm halfway to the margin, pale grayish orange (6A2) to purplish gray (17C3, 17D4) at place, with scattered small groups of dark reddish brown (9F5–6) encrustations, unchanging or slightly reddening when cut. *Stipe* central, wide, cylindrical (5.5) 6–6.5 × 2.5–4 cm; *surface* even to finely scabrous, dry, reddish to grayish gray (10B–D2) darker at the base (10D/E3), with pale violet gray (10D2) to violet brown (10F3–4) granulose squamules; *basal tomentum* little developed, white. *Stipe context* solid, orange white (5A2), yellowish orange to purplish gray (6D5, 12D/E3) virgate, slightly reddening when cut (especially near the base). *Hymenophore* tubulose, adnexed, slightly depressed around stipe; *tubes* easily separable, pale orange (5A2), 10–12 mm long halfway to the margin; *pores* at first dark violet brown (11F4) becoming brown (8F5–6). *Spore print* not recorded.

*Macrochemical reactions*: KOH: yellowish brown on pileus and stipe; NH_4_OH: yellowish brown with greenish to pinkish aura on pileus; orange with blue green aura on stipe.

*Basidiospores* from the type (immature) (5.8–) 6.6–7.5-8.4 (–8.8) × (2.9–) 3.2–3.7–4.2 (–4.4) μm, *Q* = (1.6–) 1.7–2.04–2.41 (–2.47), *N* = 55, oblong to ellipsoid with suprahilar depression, thin-walled, smooth, yellowish to brownish hyaline in water, yellowish hyaline in KOH or NH_4_OH, inamyloid. From voucher TWO1171 (9–) 9.5–11–13.6 (–14) × (3.9–) 3.9–4.4–4.9 (–5.2) μm, *N* = 50 *Q* = (2.06–) 2.18–2.51–2.98 (–3.11), narrowly ellipsoid to subcylindrical with suprahilar depression, thin-walled, smooth, yellowish to brownish hyaline in water, yellowish hyaline in KOH or NH_4_OH, inamyloid. *Basidia* 4-spored, rarely 2-spored (24–) 25–28–34 (–36) × (8–) 8–9–11 (–13) μm, with sterigmata up to 4.5 μm long, narrowly clavate to clavate, hyaline, inamyloid. *Cheilocystidia* (20–) 22–30–39 (–43) × (4–) 4–7–11 (–11) μm, frequent, narrowly fusiform to fusiform, thin-walled, hyaline. *Pleurocystidia* (29–) 29–34–40 (–40) × (5–) 5–6–7 (–7) μm, infrequent to rare, same shape as the cheilocystidia, thin-walled, hyaline. *H. trama* divergent 45–78 μm wide, with subregular mediostratum 18–26 μm wide. *Pileipellis* a palisadoderm when young becoming trichoderm, 97–128 μm thick, composed of thin-walled hyphae, with terminal cells 16–47 × 3–7.5 μm, cylindrical to subcylindrical with rounded to subacute apex somewhere, slightly yellowish to reddish pale brown in water, mostly hyaline to yellowish pale brown somewhere in KOH or NH_4_OH. *Pileus context* made of strongly interwoven hyaline hyphae, 6–16.5 μm wide, with scattered loose crystals. *Stipitipellis* a disrupted hymeniderm, 80–110 μm thick, composed of parallel hyphae, with terminal cell 13–43 × 5–9.5 μm of thin-walled, hyaline hyphae, giving rise to clusters of caulocystidia, basidiole-like cells and scarce basidia, with scattered encrustations. *Caulocystidia* (24–) 25–35–51 (–55) × (7–) 8–10–14 (–14) μm, frequent in groups, fusiform, thin-walled, hyaline. *Stipe context* composed of parallel 4- to 13.5-μm-wide hyphae, with scattered encrustations. *Clamp connections* not seen in any tissue.

*Habitat*: Solitary to gregarious, on soil, in hill evergreen forest dominated by *Castanopsis* spp., *Lithocarpus* spp. mixed with *Sh. obtusa*, *Sh. siamensis*, *D. obtusifolius* and *D*. *costatus*.

*Distribution*: Chiang Mai Province, Northern Thailand.

*Additional collections examined*: **THAILAND:** Chiang Mai Province: Mae Taeng District, Behind Wat Pah Deng, 19°06′31″N–98°44′28″E, elev. 1,070 m, July 7, 2015, Santhiti Vadthanarat, SV0098 (CMUB, BR). –ibid., Baan Mae Sae, Highway 1095 at km 55, 19°14′32.6″N–98°38′29.4″E, elev. 990 m, June 10, 2006, Osmundson, TWO1171 (MFLU, NY).

*Notes*: *S. pachypus* is characterized by the following combination of characteristics: medium-sized basidiomata; wide cylindrical stipe with granulose squamules on the stipe surface that mostly are pale violet gray to violet brown at places; pileipellis is a palisadoderm to trichoderm, subcylindrical terminal cells with rounded to subacute apex; presence of cheilocystidia and pleurocystidia. The holotype (OR0411) has oblong to ellipsoid basidiospores because it is immature. However, a mature specimen (TWO1171) has the typical shape of basidiospores, which are narrowly ellipsoid to subcylindrical with suprahilar depression. The specimen (TWO1171) was originally morphologically identified as *S*. *eximius* (Halling et al., 2012), but our more detailed examination and phylogenetic analyses indicate a new species, *S*. *pachypus*. Phylogenetically, *S. pachypus* formed a clade (clade 10) closely related to *S*. *rubinus* (clade 9), *S*. *australiensis* (clade 11), and *S*. *mucosus* (clade 12). However, all of those differ from *S. pachypus* mainly in having a narrower stipe with darker granulose squamules. The other differences are as follows: *S. rubinus* has redder basidiomata, lacks pleurocystidia, and has a trichoderm pileipellis with fusiform to broadly fusiform terminal cells with acuminate apex. *S. australiensis* has darker violet–brown pores (when young), and it has been found so far only in Australia. *S. mucosus* lacks both cheilocystidia and pleurocystidia and has a waxy to subviscid pileus, an ixotrichoderm pileipellis with fusiform to utriform terminal cells with rounded to tapering apex.

***Sutorius pseudotylopilus*** Vadthanarat, Raspé and Lumyong **sp. nov.**

MycoBank: MB838061

Figures 7F–K, 9

**FIGURE 9:**
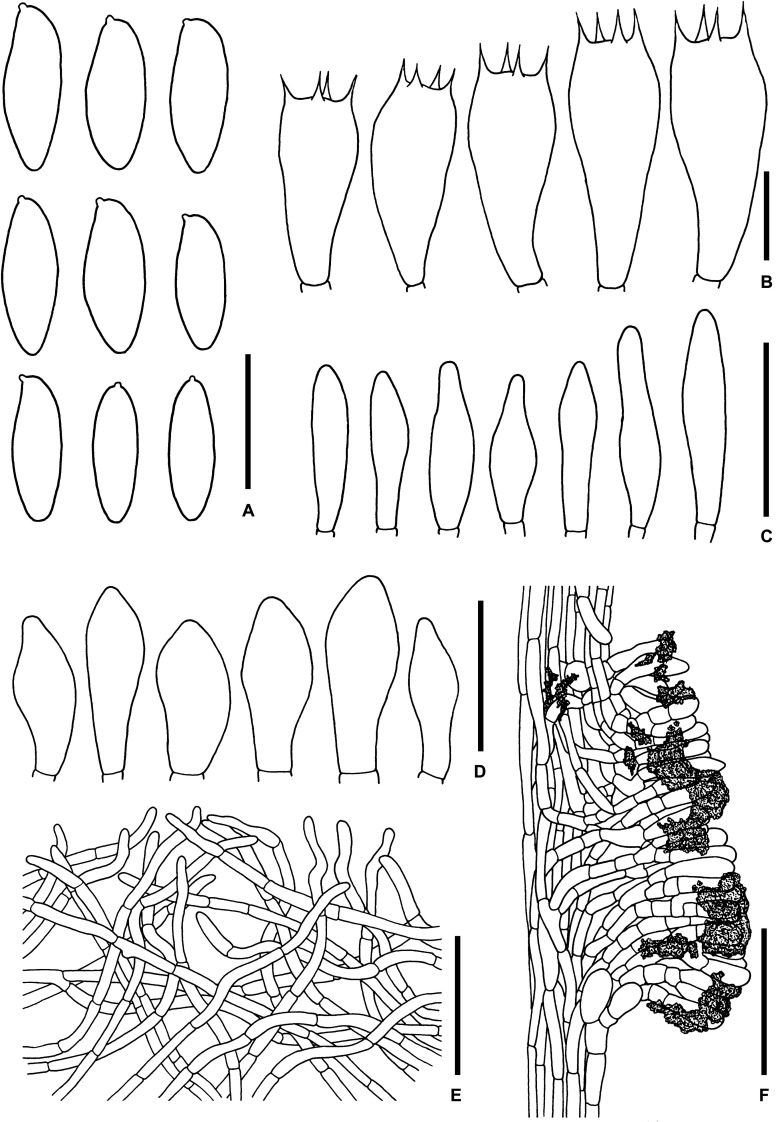
Microscopic features of *Sutorius pseudotylopilus.* **(A)** Basidiospores. **(B)** Basidia. **(C)** Cheilocystidia. **(D)** Caulocystidia. **(E)** Pileipellis. **(F)** Stipitipellis. All drawings were made from OR0378B. Scale bars: **(A,B)** = 10 μm; **(C,D)** = 25 μm; **(E,F)** = 50 μm.

*Typification*: **THAILAND**: Chiang Mai Province: Mae On District, Ban Huay Keaw community forest, 18°51′55″N–98°17′23″E, elev. 710 m, June 4, 2012, Olivier Raspé and Komsit Wisitrassameewong, OR0378B (MFLU: holotype; BR: isotype).

*Etymology*: From Greek *pseudo*–, false, and *Tylopilus* refers to the macromorphological similarity to the genus *Tylopilus*.

*Description*: *Basidiomata* medium-sized. *Pileus* 5.0–12 cm in diameter, at first convex with inflexed margin becoming planoconvex to depressed with straight margin, margin slightly exceeding (1–2 mm); *surface* even to subrugulose, dull, matted tomentose to cracked tomentose with age, patchy, grayish brown to reddish brown, brown (9–10E3–4, 7F5–6, 8F3–4). *Pileus context* 10–20 mm thick halfway to the margin, yellowish white to orange white (4A2 to 5A2), with scattered small groups of reddish brown (9F6–7) encrustations, slowly slightly reddening when cut. *Stipe* central, terete, cylindrical to slightly tapering downward, 7.5–9.5 × 1.5–2.5 cm; *surface* even, dull, dry, grayish brown to reddish brown (7–8 D/E 3–4), with grayish brown to dark brown (8–9F3–4) fine scabrous squamules; *basal tomentum* little developed, white to yellowish white. *Stipe context* solid, orange white (5A2) near the cap to yellowish white (4A2) to greenish white (3B3) at the base, grayish brown to reddish brown (7–8 D/E 3–4) virgate, with scattered brownish gray (8–10E2) or reddish brown (9D4, 9F4–5) encrustations, slowly slightly reddening when cut. *Hymenophore* tubulose, narrowly adnexed to adnate, subventricose to ventricose, sometimes slightly depressed around the stipe; *tubes* easily separable, brown to reddish brown (7–8E4–5), 7–15 mm long halfway to the margin, unchanged or slightly reddening when cut; *pores* 0.4–0.8 mm wide at midradius, mostly roundish, at first dark purple (11F3–4) becoming dark brow to brown (8–9F5–6 to 7F5–6) with age. *Odor* not observed *taste* slightly acid. *Spore print* not observed.

*Macrochemical reactions*: KOH: yellowish then brownish on the cap, stipe, and pileus context; yellowish or greenish on stipe context; yellowish on hymenium. NH_4_OH: yellowish red on the pileus; yellowish to brownish on pileus context, stipe, stipe context, and hymenium.

*Basidiospores* [168/3/3] (9.3–) 10.2–11.9–14 (–15.5) × (3.4–) 3.9–4.3–5 (–5) μm, *Q* = (2.18–) 2.4–2.78–3.24 (–3.82). From the type (9.3–) 9.6–11.1–13.1 (–14.1) × (3.4–) 3.8–4.2–4.5 (–4.7) μm, *Q* = (2.18–) 2.25–2.66–3.44 (–3.82), *N* = 53, narrowly ellipsoid to subcylindrical with slight of suprahilar depression, thin-walled, smooth, brownish to yellowish hyaline in water, yellowish hyaline in KOH or NH_4_OH, inamyloid. *Basidia* 4-spored, (19–) 20–24–28 (–28) × (9–) 9–10–11 (–11) μm, with sterigmata up to 4 μm long, clavate, hyaline, inamyloid. *Cheilocystidia* (21–) 21–24–33 (–33) × (4–) 4–6–7 (–7) μm, frequent, mostly cylindrical to fusiform with rounded to tapering apex, thin-walled, hyaline. *Pleurocystidia* not seen. *H. trama* divergent to boletoid 30–44 μm wide, with regular to subregular mediostratum 7.5–23 μm wide. *Pileipellis* an intricate to flattened trichoderm, 85–130 μm thick, made of loosely to moderately interwoven hyphae, thin-walled, mostly hyaline to yellowish pale brown in KOH or NH_4_OH, with slightly curly cylindrical terminal cells 14–46 × 3.5–6 μm with rounded apex, with scattered loose crystals. *Pileus context* made of strongly interwoven hyaline hyphae, 5.5–8.5 μm wide, with scattered loose crystals. *Stipitipellis* a disrupted palisadoderm, 90–110 μm thick, with clusters of basidiole-like cells 9–25 × 4–9 μm of thin-walled, mostly hyaline to pale yellowish brown hyphae, subtended by short chains of 3–4 (sub) isodiametric cells, with scattered encrustations. *Caulocystidia* (18–) 18–25–30 (–30) × (7–) 7–10–12 (–12) μm, infrequent, fusiform to broadly fusiform, thin-walled, hyaline. *Stipe context* composed of parallel, 4- to 11-μm-wide hyphae, with scattered encrustations. *Clamp connections* not seen in any tissue.

*Habitat*: Solitary or fasciculate on soil, in dipterocarp forest dominated by *D. obtusifolius*, *D*. *tuberculatus*, *Sh. obtusa*, and *Sh*. *siamensis* and a few *Hopea* sp., as well as *Castanopsis* spp.

*Distribution*: Chiang Mai Province, Northern Thailand.

*Notes*: *Sutorius pseudotylopilus* is characterized by the following combination of characteristics: medium-sized basidiomata, purplish to reddish brown basidiomata when young; lack of pleurocystidia; pileipellis constituted by an intricate to flattened trichoderm with cylindrical, slightly curly terminal cells with rounded apex. Phylogenetically, the three *S*. *pseudotylopilus* specimens formed a clade (clade 4) sister to three undescribed *Sutorius* species: clade 1 contains three specimens from China (HKAS59657, HKAS52672, and HKAS56291); *Sutorius* clade 2 contains another specimen from China (HKAS56291), and *Sutorius* clade 3 contains one specimen (TWO986) from Costa Rica in Central America. The closest described species is *S*. *eximius* (American species) in clade 5. However, *S*. *eximius* morphologically differs from *S*. *pseudotylopilus* by the following characteristics: stipe context unchanging when injured; presence of pleurocystidia (Singer, 1947; Smith and Thiers, 1971; Halling et al., 2012). Moreover, based on our latest phylogenetics analyses with more *Sutorius* exemplars, we hypothesize that *S*. *eximius* is restricted to North America (clade 5).

*Additional collections examined*: **THAILAND:** Chiang Mai Province: Mae On District, Ban Huay Kaew community forest, 18°51′56″N–99°17′24″E, elev. 700 m, June 1, 2017, Santhiti Vadthanarat, SV0401 (CMUB, BR); –ibid., 18°51′55″N–99°17′24″E, elev. 700 m, May 14, 2018, Santhiti Vadthanarat, SV0415 (CMUB, BR).

***Sutorius rubinus*** Vadthanarat, Raspé and Lumyong **sp. nov.**

MycoBank: MB838062

Figures 10A–F, 11

**FIGURE 10:**
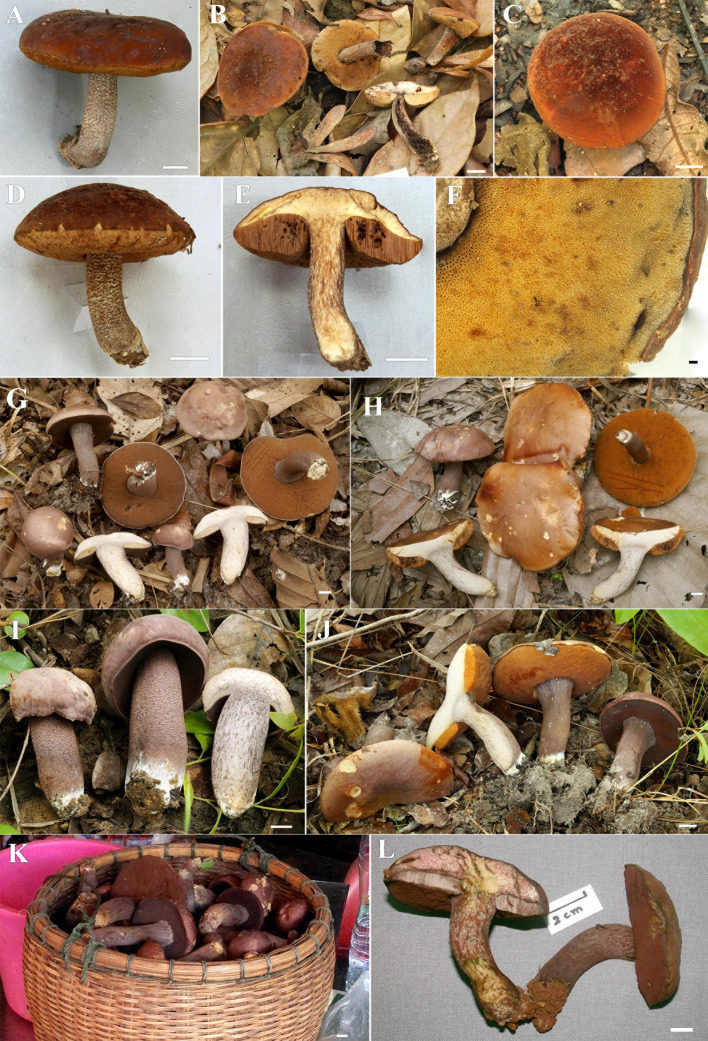
**(A–F)** Fresh basidiomata of *Sutorius rubinus* (**A**: OR0379, **B**: OR0403, **C–E**: OR0409, **F**: pores surface when young in OR1255). **(G–K)** Fresh basidiomata of *Sutorius ubonensis*. (**G**: SV0029, **H**: SV0032, **I**: SV0203, **J**: SV0313, **K**: SV0353 a collection from a local market in Ubon Ratchathani Province). **(L)** Fresh basidiomata of *Sutorius vellingae* specimen voucher ECV3603. Scale bar: **(A–L)** = 1 cm.

**FIGURE 11:**
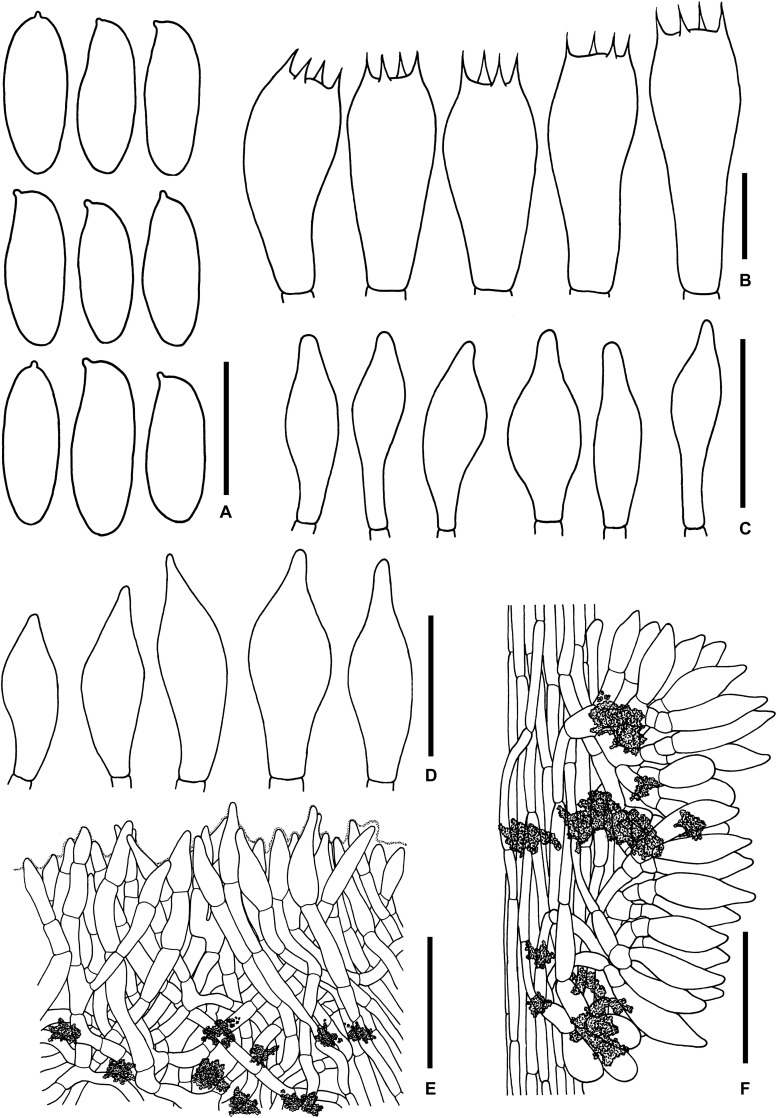
Microscopic features of *Sutorius rubinus*. **(A)** Basidiospores. **(B)** Basidia. **(C)** Cheilocystidia. **(D)** Caulocystidia. **(E)** Pileipellis. **(F)** Stipitipellis. All drawings were made from OR0403. Scale bars: **(A,B)** = 10 μm; **(C,D)** = 25 μm; **(E,F)** = 50 μm.

*Typification*: **THAILAND:** Chiang Mai Province: Mae Taeng District, Pha Deng village, 19°06′38″N–98°44′32″E, elev. 1,055 m, June 7, 2012, Olivier Raspé and Komsit Wisitrassameewong, OR0403 (MFLU: holotype; BR: isotype).

*Etymology*: From Latin rubinus, red, referring to basidiomata color.

*Description*: *Basidiomata* medium-sized. *Pileus* 4.5–7.5 cm in diameter, convex to planoconvex, with slightly deflexed, becoming straight margin, which is slightly exceeding (1–2 mm) especially when young; *surface* at first subrugulose to rugose becoming scabrous to subrugulose at center, tomentose, reddish dark brown (9F4–6) becoming reddish dark brown (8F5–8) to reddish brown (8D/E6–8) with age, gradually paler to the margin. *Pileus context* 5–10 mm thick halfway to the margin, orange white (5A2), with scattered small groups of reddish brown (8–9D4–5) encrustations. *Stipe* central, terete to slightly compressed, cylindrical to subclavate or tapering downwards, sometimes with subbulbous base, 5–6.5 × 0.8–2.2 cm; *surface* finely scabrous, reddish gray to grayish brown (8 D/E 2–3) densely covered with reddish brown to dark brown (8F4–5) granulose squamules; *basal tomentum* white to off-white. *Stipe context* solid, orange white to pinkish white (5A2 to 7A2), reddish to grayish brown to dark brown (8D–F3–5) virgate, with scattered small groups of reddish brown (8–9D4–5) encrustations, unchanged or slightly reddening when cut. *Hymenophore* tubulose, adnexed to narrowly adnexed, ventricose, slightly depressed around the stipe; *tubes* dull yellowish white (3A3–4) when young becoming orange-brown (7E7) with age, easily separable, 6–11 mm long halfway to the margin; *pores* 0.3–0.5 mm wide at midradius, mostly roundish, at first grayish orange (5D3–4) becoming orange to pale orange (5A3–4 to 6B6–7) with age, with scattered small groups of reddish dark brown (8F3–4) encrustations. *Odor* and *taste* not observed. *Spore print* not observed.

*Macrochemical reactions*: KOH: dark brown to black on the stipe, reddish on the hymenophore, negative on the pileus, pileus context, and stipe context.

*Basidiospores* [168/3/3] (8.7–) 9.6–10.8–12.1 (–13.1) × (3.5–) 3.9–4.3–4.8 (–5.1) μm, *Q* = (2.03–) 2.2–2.5–2.82 (–2.96). Form the type (8.7–) 9.3–10.5–12.1 (–12.3) × (3.5–) 3.9–4.4–4.9 (–5.1) μm, *Q* = (2.03–) 2.06–2.4–2.73 (–2.81), *N* = 57, narrowly ellipsoid to subcylindrical with slightly suprahilar depression, thin-walled, smooth, yellowish hyaline in KOH or NH_4_OH, inamyloid. *Basidia* 4-spored (23–) 23–27–31 (–31) × (10–) 10–11–13 (–13) μm, with sterigmata up to 4 μm long, clavate, hyaline, inamyloid. *Cheilocystidia* (20–) 20–26–32 (–32) × (5–) 5–7–8 (–8) μm, frequent, narrowly fusiform to fusiform with subacute apex, thin-walled, hyaline. *Pleurocystidia* not seen. *H. trama* divergent, 42–80 μm wide, with regular to subregular mediostratum 8–27 μm wide. *Pileipellis* an intricate trichoderm to slightly gelatinized intricate trichoderm, 60–110 μm thick, made of densely interwoven, thin-walled, smooth, hyaline hyphae 4–12 μm wide, with terminal cells 11–40 × 5–12 μm, fusiform to broadly fusiform with acuminate apex, slightly yellowish to reddish pale brown in water, mostly hyaline to yellowish pale brown in KOH or NH_4_OH. *Pileus context* made of strongly interwoven hyphae, 6–18 μm wide, thin-walled, with scattered loose crystals. *Stipitipellis* a disrupted hymeniderm, 90–120 μm thick, composed of parallel hyphae, thin-walled, with terminal cells 19–30 × 11–14 μm, giving rise to clusters of caulocystidia and basidiole-like cells, with scattered encrustations. *Caulocystidia* (23–) 23–31–40 (–40) × (8–) 8–12–18 (–18) μm, frequent, fusiform with subacute apex, thin-walled, hyaline. *Stipe context* composed of parallel, 8- to 12-μm-wide hyphae, with scattered encrustations. *Clamp connections* not seen in any tissue.

*Habitat*: Solitary to gregarious, on soil, in evergreen hill forest dominated by *Castanopsis* spp., *Lithocarpus* spp. mixed with *D. tuberculatus*, *D*. *obtusifolius*, *Sh. obtusa*, and *Sh*. *siamensis*.

*Distribution*: Chiang Mai Province, Northern Thailand

*Notes*: *S. rubinus* is characterized by the following combination of characteristics: medium-sized basidiomata, dark reddish brown to reddish brown; lack of pleurocystidia; pileipellis a trichoderm with fusiform to broadly fusiform terminal cells with acuminate apex.

Phylogenetically, all *S*. *rubinus* specimens formed a clade (clade 9) close to three *Sutorius* species including *S. pachypus* (clade 10), *S*. *australiensis* (clade 11), and *S. mucosus* (clade 12). Nevertheless, they are morphologically different from *S*. *rubinus* as follows: *S. pachypus* has on average wider (2.5–4 cm wide) stipe and has pleurocystidia, and the pileipellis is a palisadoderm composed of subcylindrical with rounded to subacute apex. *S. australiensis* has dark violet brown pores when young, a trichoderm pileipellis composed of elongated to cylindrical elements with obtuse apex and is found only in Australia. *S. mucosus* has a waxy to subviscid pileus with an ixotrichoderm forming the pileipellis composed of gelatinized hyphae, with fusiform to utriform terminal cells with rounded to subacute apex.

*Additional collections examined*: **THAILAND:** Chiang Mai Province: Mae On District, Ban Huay Keaw community forest, 18°51′55″N–99°17′23″E, elev. 710 m, June 4, 2012, Jie Chen and Olivier Raspé, OR0379 (MFLU12-0231); Mae Taeng District, Pha Deng village, 19°06′38″N–98°44′32″E, elev. 1,055 m, June 7, 2012, Olivier Raspé and Komsit Wisitrassameewong, OR0409 (MFLU12-0261); ibid., June 24, 2016, Olivier Raspé, OR1255 (CMUB, BR).

***Sutorius ubonensis*** Vadthanarat, Raspé and Lumyong **sp. nov.**

MycoBank: MB838066

Figures 10G–K, 12

**FIGURE 12:**
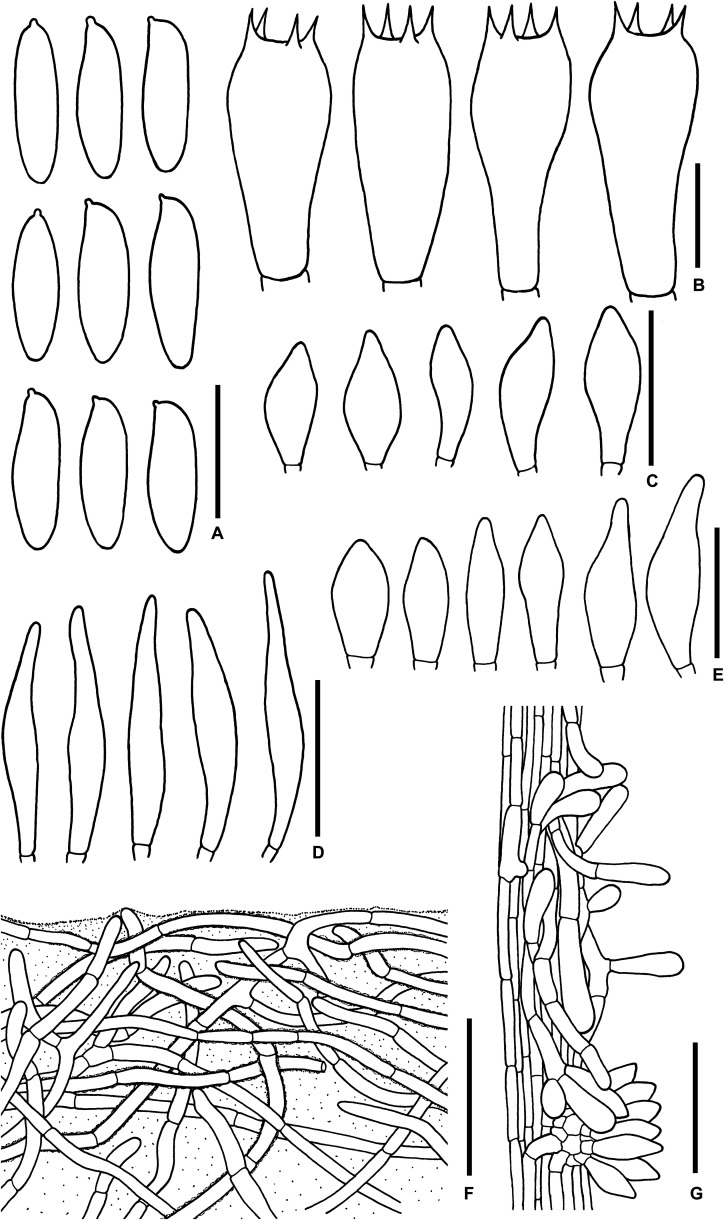
Microscopic features of *Sutorius ubonensis*. **(A)** Basidiospores. **(B)** Basidia. **(C)** Cheilocystidia. **(D)** Pleurocystidia. **(E)** Caulocystidia. **(F)** Pileipellis. **(G)** Stipitipellis. Drawings **(A–D)** were made from SV0032, **(F,G)** were made from SV0029. Scale bars: **(A,B)** = 10 μm; **(C–E)** = 25 μm; **(F,G)** = 50 μm.

*Typification*: **THAILAND:** Ubon Ratchathani Province: Trakan Phuet Phon District, Ban Huay Fai community forest, 15°32’44”N–105°10’15”E, elev. 160 m, July 11, 2014, Santhiti Vadthanarat, SV0032 (CMUB: holotype; BR: isotype).

*Etymology*: ubonensis refers to Ubon Ratchathani Province where the species was found.

*Description*: *Basidiomata* medium-sized. *Pileus* (5.5) 8.0–10.5(12) cm in diameter, at first hemispherical to convex with straight to slightly inflexed margin, becoming convex to planoconvex to depressed with straight to slightly reflexed margin, margin slightly exceeding (1–2 mm), elastic; *surface* even to subrugulose, dull, subviscid when wet, minutely tomentose, at first violet to violet brown (10E4) to dark brown (9F5–6), later become reddish gray (10B2) to dull red or brownish gray (10C/D3) at the center, getting paler to brownish gray to (8C2) near the margin, sometimes patchy of reddish gray (8–9C/E3–4) purplish brown (10F4–5) or brown (6E6). *Pileus context* 10–18 mm thick halfway to the margin, yellowish white to orange white (4A2 to 5A2), with scattered small groups of reddish brown (9D4–6) encrustations, unchanged. *Stipe* central, terete to subcompressed, cylindrical to subclavate or tapering downwards (4.8) 5.2–8.5 × 1.8–3.2 cm; *surface* even to finely scabrous, slightly venose to rimose near the cap, dull to slightly shiny, dry, purplish gray to brownish gray (9–10C/E2), scattered of reddish brown to dark brown (9F4–5) granulose squamulose; *basal tomentum* off-white to yellowish white. *Stipe context* solid, slightly fibrous, yellowish white (4A2 to 5A2) to slightly purplish gray (15B/C2) near the stipitipellis, grayish brown to purplish brown (8–9E/F3–5) virgate, with scattered brownish gray (8–10E2) or reddish brown (9D4 to 9F4–5) encrustations, unchanged. *Hymenophore* tubulose, adnate, subventricose to ventricose; *tubes* easily separable, at first purplish to yellowish gray (8C2 to 6B3 to 5B3–4) becoming yellowish orange to brownish orange (5B3–4 to 6B3–4) with age, 8–13 mm long halfway to the margin; *pores* 0.3–0.8 mm wide at midradius, mostly roundish, slightly elongated near the stipe, subregular especially when young, dark brown to purplish dark brown (9F4–6 to 10F3–5) at first, become brown (7F5–6) to orange brown (6E4–5, 7E5–6) with age. *Odor* fungoid to slightly acidulate. *Taste* mild to slightly acid. *Spore print* brownish orange (7C3) to dark brown (7F6–7) in mass.

*Macrochemical reactions*: KOH: rapidly slightly greenish and then yellow on pileus and stipe; yellowish on pileus context, stipe context, and hymenium. NH_4_OH: yellowish with greenish to purplish aura on the cap and stipe; yellowish on pileus context, stipe context, and hymenium.

*Basidiospores* [172/3/2] (8.7–) 9.8–12–14.7 (–16.8) × (3.1–) 3.4–4–4.4 (–4.7) μm *Q* = (2.61–) 2.42–3.01–3.63 (–3.93). From the type (10.1–) 11.1–13.4–16.6 (–16.8) × (3.6–) 3.7–4.1–4.6 (–4.7) μm, *Q* = (2.68–) 2.74–*3.27*–3.81 (–3.93), *N* = 57, narrowly ellipsoid to subcylindrical with slightly suprahilar depression, thin-walled, smooth, brownish to yellowish hyaline in water, yellowish hyaline in KOH or NH_4_OH, inamyloid. *Basidia* 4-spored (19–) 19–22–25 (–25) × (9–) 9–10–11 (–11) μm, with sterigmata up to 4 μm long clavate, hyaline, inamyloid. *Cheilocystidia* (19–) 19–23–28 (–28) × (6–) 6–8–10 (–10) μm, frequent, fusiform, thin-walled, hyaline. *Pleurocystidia* (34–) 35–40–47 (–47) × (5–) 5–6–7 (–7) μm, frequent, narrowly fusiform, thin-walled, hyaline. *H. trama* divergent to boletoid, 57–78 μm wide, with 18–42 μm wide of regular mediostratum. *Pileipellis* a tomentum to slightly gelatinized tomentum, 89–118 μm thick, composed of moderately interwoven, hyaline, thin-walled hyphae, with terminal cells 19–57 × 5–8.5 μm, cylindrical with rounded apex, some hyphae were covered with mucus on the wall, at places with scattered of loose crystals. *Pileus context* made of strongly interwoven hyphae, 8.5–13 μm wide, at places with scattered of loose crystals. *Stipitipellis* hyphae vertically oriented (80–95 μm thick), pointing out of terminal cell 10–35 × 4–12.5 μm of thin-walled with rounded apex, hyaline hyphae, giving rise to clusters of *caulocystidia*, which (23–) 23–27–35 (–35) × (7–) 7–10–13 (–13) μm, frequent in group, fusiform, thin-walled, hyaline, with somewhere scattered of loose crystals especially near the surface. *Stipe context* composed of parallel, 4.5- to 8-μm-wide hyphae, with scattered small loose crystals. *Clamp connections* not seen in any tissue.

*Habitat*: Solitary to gregarious, sometimes fasciculate, on soil in dipterocarp forest dominated by *D. obtusifolius*, *D*. *tuberculatus*, *Dipterocarpus intricatus*, *Sh. obtusa*, and *Sh*. *siamensis*.

*Distribution*: Ubon Ratchathani Province, Northeastern Thailand.

*Notes*: *S. ubonensis* is characterized by the combination of following characteristics: medium sized basidiomata, purplish gray when young becoming purplish to reddish brown with age; unchanged context; pileipellis a tomentum to slightly gelatinized tomentum, with cylindrical terminal elements with rounded apex; found in dipterocarp forest in Northeastern Thailand. Morphologically, *S. ubonensis* is superficially similar to *S. eximius* in macro-characters especially when young. Both species are also similar in some microscopic characters including the presence and shape of cheilocystidia, pleurocystidia, and caulocystidia, as well as the shape of elements on pileipellis. However, they are different in pileipellis structure, with *S. eximius* having a trichoderm, whereas *S. ubonensis* has a tomentum to slightly gelatinized tomentum. The two species also occur on different continents, with *S*. *ubonensis* being found in Ubon Ratchathani Province, Northeastern Thailand, Southeast Asia, whereas *S. eximius* is found in North America (Singer, 1947; Smith and Thiers, 1971; Halling et al., 2012).

Phylogenetically, *S. ubonensis* (clade 7) was clusters in a poorly supported, long-branch, sister to *S*. *obscuripellis* (clade 8) from Chiang Mai Province, Thailand, and *S*. *subrufus* (clade 6) from China. However, these two species differ from *S. ubonensis* as follows: *S*. *obscuripellis* has smaller basidiomata and is also paler, especially when young; lacks pleurocystidia; and has a trichoderm pileipellis composed of slightly dark to dark hyphae, with cylindrical terminal cells with subacute to rounded apex. So far, S. *obscuripellis* has been found only in Chiang Mai Province. *S. subrufus* also has paler basidiomata; paler pores, especially when young, which are pale brown to brown to pale reddish brown; stipe surface and context turn reddish when injured; a trichoderm pileipellis, composed of rather vertically arranged, with clavate or subclavate terminal cells, with obtuse apex; found in the forests dominated by Fagaceae trees, including *Lithocarpus* spp. (Chai et al., 2019). So far, *S. ubonensis* is the only *Sutorius* species found in Ubon Ratchathani Province, Northeastern Thailand. It occurs in dry dipterocarp forest at the lowest elevation (about 150–175 m) compared to the other *Sutorius* species.

*Additional collections examined*: **THAILAND:** Ubon Ratchathani Province: Trakan Phuet Phon District, Ban Huay Fai community forest, 15°32′43.58″N–105°10′16″E, elev. 160 m, July 11, 2014, Santhiti Vadthanarat, SV0029 (CMUB, BR); –ibid., 15°32′42”N–105°10′16″E, elev. 175 m, July 24, 2016, Santhiti Vadthanarat, SV0313 (CMUB, BR); –ibid., 15°32′42”N–105°10′16″E, elev. 160 m, July 15, 2017, Santhiti Vadthanarat, SV0405 (CMUB, BR); –ibid., 15°32′43”N–105°10′17″E, elev. 160 m, July 15, 2017, Santhiti Vadthanarat, SV0407 (CMUB, BR); –ibid., 15°32′43”N–105°10′15″E, elev. 175 m, July 15, 2017, Santhiti Vadthanarat, SV0410 (CMUB, BR); Sri Suk Village, Khok Tam Lae community forest, 15°35′46″N–105°06′35″E, elev. 150 m, August 6, 2015, Santhiti Vadthanarat, SV0203 (CMUB, BR); Trakan Phuet Phon market, September 22, 2016, Santhiti Vadthanarat, SV0353 (CMUB, BR).

***Sutorius vellingae*** Vadthanarat, Raspé and Lumyong **sp. nov.**

MycoBank: MB838065

Figures 10L, 13

**FIGURE 13:**
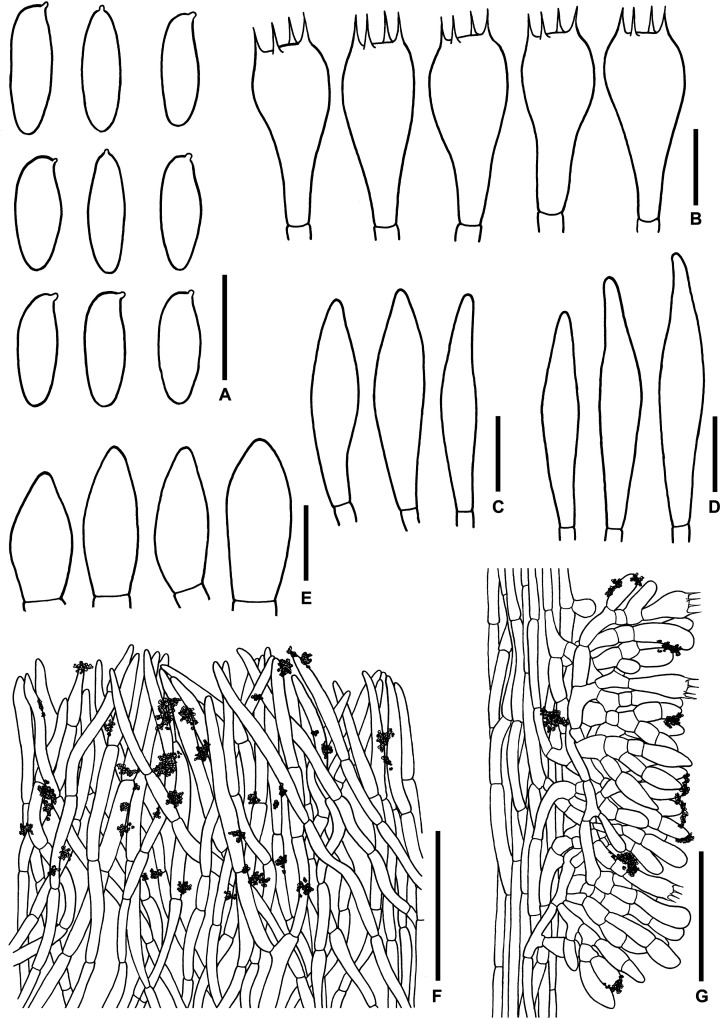
Microscopic features of *Sutorius vellingae*. **(A)** Basidiospores. **(B)** Basidia. **(C)** Cheilocystidia. **(D)** Pleurocystidia. **(E)** Caulocystidia. **(F)** Pileipellis. **(G)** Stipitipellis. All drawings were made from ECV3603. Scale bars: **(A–E)** = 10 μm; **(F,G)** = 50 μm.

*Typification*: **THAILAND:** Chiang Mai Province: Mae Taeng District, Baan Mae Sae, Highway 1095 at km 55, 19°14′32.6″N–98°38′29.4″E, elev. 990 m, July 4, 2007, Else C. Vellinga, ECV3603 (MFLU: holotype, NY isotype).

*Etymology*: Named after Else C. Vellinga, who collected the type specimen.

*Description*: *Basidiomata* medium-sized. *Pileus* 7 × 5.1 cm, convex, margin slightly exceeding (1 mm); *surface* even to subrugulose, dark brown (8F6–7) with some purple (10F7) shining through at places; *pileus context* 12–13 mm thick halfway to the margin, yellowish white (2A2), with scattered small groups of reddish brown (9E/D7–8) encrustations. *Stipe* central, curved, cylindrical to slightly compressed, 6.8 × 1.6 cm; *surface* even to finely scabrous, with some veins on the upper part, dull to slightly shiny, reddish gray to grayish brown (9C2–3), scattered with reddish brown (10F8) granulose squamules to purplish dark brown (9–10F4–6) granulose squamules near the base; *basal tomentum* little developed, yellowish white; *stipe context* solid, marmorate to virgate, yellowish white (2–3A2) and brownish red (10E6–7) to violet brown (10F6–7), with scattered small groups of reddish brown (9E/D7–8) encrustations. *Hymenophore* tubulose, adnate; *tubes* pinkish white (9A2), 7 mm long halfway to the margin; *pores* fine, subregular, violet brown (10F8). *Odor* fungoid. *Taste* not distinctive. *Spore print* not observed.

*Basidiospores* (9–) 9.5–10.4–11.1 (–11.5) × (3.6–) 3.8–4.2–4.6 (–4.8) μm, *Q* = (2.22–) 2.22–2.52–2.82 (–2.93), *N* = 65, narrowly ellipsoid to subcylindrical with slight suprahilar depression, thin-walled, smooth, brownish to yellowish hyaline in water, yellowish hyaline in KOH or NH_4_OH, inamyloid. *Basidia* 4-spored (19–) 19–24–28 (–28) × (10–) 10–11–12 (–12) μm, with sterigmata up to 4.5 μm long clavate, hyaline, inamyloid. *Cheilocystidia* 28–29 × 5–8 μm, rare, narrowly fusiform to fusiform, thin-walled, hyaline. *Pleurocystidia* 29–35 × 6–7 μm, rare, narrowly fusiform to fusiform, thin-walled, hyaline. *H. trama* divergent, 36–77 μm wide, with 10–31 μm wide of subregular mediostratum. *Pileipellis* a trichoderm, 110–140 μm thick, thin-walled hyphae, composed of cylindrical terminal cells 23–61 × 4–7 μm, with subacute to acute apex, slightly yellowish hyaline in water, slightly greenish to yellowish hyaline in KOH or NH_4_OH, with scattered yellowish-brown parietal encrustations. *Pileus context* made of moderately interwoven hyaline hyphae, 4–10 μm wide, with scattered reddish brown loose crystals in water or NH_4_OH. *Stipitipellis* a disrupted hymeniderm, 130–175 μm thick, composed of parallel hyaline hyphae anastomosing at places and terminal cells 13–19 × 7–8 μm, thin-walled, giving rise to clusters of basidiole-like cells and caulocystidia, with scattered loose crystals. *Caulocystidia* (17–) 17–21–30 (–30) × (7–) 7–9–11 (–11) μm, frequent in groups, fusiform to broadly fusiform, thin-walled, hyaline. *Stipe context* composed of parallel 4.5- to 9-μm-wide hyphae, with scattered loose crystals. *Clamp connections* not seen in any tissue.

*Habitat*: Solitary under *Castanopsis* and *Lithocarpus* trees.

*Distribution*: Chiang Mai Province, Northern Thailand.

*Notes*: The *Sutorius* specimen voucher ECV3603 was first recognized as a taxon discrete from *S*. *eximius* and *S*. *australiensis* in the original publication of *Sutorius*, based on phylogenetic analysis of a combined *LSU* + *tef*1 dataset (Halling et al., 2012). However, it was not described as a new species at that time because of the solitary nature of the collection. In this study, that same specimen was further studied and is hypothesized to be a new species, *S. vellingae*. Based on that single collection, *S. vellingae* is characterized by the combination of following characteristics: marked violet brown pore color; pileipellis trichoderm composed of cylindrical terminal cells with subacute to acute apex; rare, narrowly fusiform to fusiform cheilocystidia and pleurocystidia; and its very distinct, isolated phylogenetic position within *Sutorius*.

### Key to *Sutorius* Species Worldwide

**1** Distribution in the Americas or Australia.............................. **2**
**1′** Distribution in Asia.................................................................. **3**
**2** Found in the Americas............................................. ***S*. *eximius***
**2′** Found in Australia........................................... ***S*. *australiensis***
**3** Pileipellis a palisadoderm or trichoderm to intricate trichoderm..................................................................................... **4**
**3′** Pileipellis a tomentum to slightly gelatinized tomentum composed of cylindrical terminal elements; basidiomata medium sized; at first pileus and pores purple to purplish brown to dark brown becoming purplish to reddish brown.......................................................................... ***S. ubonensis***
**4** Pileipellis composed of wide cylindrical hyphae, with terminal cells fusiform to broadly fusiform or utriform (5–12 μm wide)............................................................................ **5**
**4′** Pileipellis composed of cylindrical hyphae, with narrow cylindrical to cylindrical terminal cells (3–7 μm wide)......... **7**
**5** Pileipellis strongly gelatinized, composed of slimy hyphae, with terminal cells fusiform to utriform with rounded to subacute apex............................................................... ***S*. *mucosus***
**5′** Pileipellis not to slightly gelatinized, composed of hyphae with terminal cells fusiform to broadly fusiform with acuminate or tapering apex......................................................... **6**
**6** Basidiomata reddish dark brown to reddish brown; pores grayish orange at first; pileipellis with fusiform to broadly fusiform terminal cells, with acuminate apex........... ***S. rubinus***
**6′** Basidiomata dark lilac to purplish to reddish brown, pores lilac gray at first; pileipellis with fusiform terminal cells, with tapering apex....................................................... ***S. maculatoides***
**7** Pileipellis an intricate trichoderm........................................... **8**
**7′** Pileipellis a palisadoderm or trichoderm composed of almost vertically arranged hyphae.............................................. **9**
**8** Basidiomata small- to medium-sized, pileus 3.5–6 cm in diam.; pileipellis composed of cylindrical hyphae, terminal cells cylindrical with subacute to rounded apex, with scattered small parietal encrustations, brown in KOH..... ***S. obscuripellis***
**8′** Basidiomata medium-sized, pileus 5–9.5 cm in diam.; pileipellis composed of cylindrical hyphae with terminal cells cylindrical and slightly curly, mostly hyaline to yellowish pale brown in KOH ............................................... ***S. pseudotylopilus***
**9** Terminal cells of the pileipellis cylindrical with obtuse to rounded apex; pleurocystidia frequent.................................... **10**
**9′** Terminal cells of the pileipellis are cylindrical with tapering to acute apex; pleurocystidia rare.............................. ***S. vellingae***
**10** Basidiomata with wide cylindrical stipe 5.5–6.5 × 2.5–4 cm; stipe surface unchanging when injured ................. ***S. pachypus***
**10′** Basidiomata with subcylindrical stipe 6–10 × 1–2.2 cm; stipe surface usually reddening when injured ........ ***S*. *subrufus***

## Discussion

*Sutorius* species are morphologically roughly similar to each other by their chocolate to reddish brown or purplish brown basidiomata, with granulose squamules scattered or transversely scissurate scales on the stipe surface. For this reason, macromorphological features are often not enough for species identification. Observation of microscopic characters is needed. The dimensions and *Q*-value of the basidiospores are the characters commonly used to differentiate species in the Boletaceae. In *Sutorius*, however, the dimensions and *Q*-value of the basidiospores seem to be poor characters for species delineation, because spore size and *Q*-value, especially the length of the basidiospores, can vary greatly within the same species and sometimes within the same individual. For example, in this study, the basidiospore length of *S*. *ubonensis* varied from 10.1 to 16.8 μm in the same basidioma, and from 8.7 to 16.8 μm between different collections. Therefore, in this study, the dimensions and *Q*-value of basidiospores were not used to differentiate species. On the basis of this study and previously published data, the appropriate set of characters for species identification of *Sutorius* is the combination of macromorphological features including basidiomata size, color, and stipe surface, and microscopic characters such as the arrangement and shape of terminal cells of the pileipellis, and the presence/absence or frequency of hymenophoral cystidia. Those characters mentioned above show little intraspecific variability and allow a clear-cut species delimitation. Some of the new species, namely, *S*. *mucosus*, *S*. *obscuripellis*, and *S*. *vellingae*, were described based on a single collection or basidiomata. However, all morphological and phylogenetic evidence was reliable and compelling enough to describe those species as new. During our survey in the North of Thailand, starting in 2012, we could not get more collections of those species, which suggests that those species are uncommon or rare. More collections of those species would be useful to better describe and understand the intraspecific genetic and morphological variability.

In the literature, *Sutorius eximius* has also been reported from Thailand (as *T. eximius*) (Chantorn et al., 2007; Chandrasrikul et al., 2008; Vadthanarat, 2009; Vadthanarat et al., 2010). Those reports were based on morphological characters only. Our phylogenetic analyses on Thai collections showed that they belong to eight different species, but none of them belongs in *S*. *eximius*. The phylogenetically closest species to *S*. *eximius* (voucher REH9400, from United States) was *S*. *pseudotylopilus*, but it differs morphologically and ecologically from *S*. *eximius* (see in *S*. *pseudotylopilus* notes). Morphologically, the most similar species is *S*. *ubonensis*, which was previously reported as *T*. *eximius* from Ubon Ratchathani Province (Vadthanarat, 2009; Vadthanarat et al., 2010). Another report of *T*. *eximius* was from Nam Nao national park, Northeastern Thailand (Chantorn et al., 2007). We could not obtain the specimen for this study, but it can reasonably be assumed that, again, it belongs to a different species, other than *S. eximius*.

In Northeastern Thailand, *S. ubonensis* is one of the wild edible boletes that is collected and consumed by local people. It is called “Hed Pheung Khao Kam” or “Hed Pheung E-Dam,” which means purple or blackish bolete. The price of *S. ubonensis* is usually about 200–300 THB/kg. Local people usually cook it by steaming or boiling over a long time period. They also believe that if it is not properly cooked, it might be poisonous.

In this study, a total of seven new *Sutorius* species and one new combination are proposed from Thailand and Southeast Asia. Before this study, only three *Sutorius* species were described worldwide, with one species from each of three continents, the Americas, Australia, and Asia (Halling et al., 2012; Chai et al., 2019). At present, 11 *Sutorius* species are known including the eight additional species in this study. In addition to those 11 species, our phylogeny (Figure 1) resolved six more terminal clades, which are phylogenetically distinct from all the described species. Among the six species, three are from Africa (Togo, Zimbabwe, and Burundi; clades 14, 15, and 16), two are from Asia (China; clades 1 and 2), and another was from Central America (Costa Rica; clade 3). Moreover, in previous studies, some undescribed *Sutorius* species were also phylogenetically evidenced from China, Zambia, and Indonesia, but have not yet been described (Halling et al., 2012; Chai et al., 2019). In South America, several *Sutorius* collections from Guyana were also morphologically identified and reported as *T*. *eximius* (Fulgenzi et al., 2007). Unfortunately, those latter collections were not added in our phylogeny because there were no sequences available in GenBank compatible with our phylogenetic study, and we have not been able to obtain the specimens. They might correspond as allies to some of the newly described species in this study. It is noteworthy that, so far, there is not any *Sutorius* collection known from Europe (Fulgenzi et al., 2007; Halling et al., 2012; Chai et al., 2019). Phylogenetically, the African specimens clustered together in a well-supported subclade within *Sutorius*, sister to another highly supported subclade comprising American, Australian, and Asian specimens with the exception of *S*. *vellingae* (ECV3603). *S. vellingae* (ECV3603) was not in the same clade with the other Asian species, but formed an isolated terminal clade, sister to all other *Sutorius* species. The pattern of two well-supported clades of African specimens sister to the others was also observed in the genus *Pulveroboletus* (Badou et al., 2018).

This study has refined the original concept of *Sutorius* and its members proposed by Halling et al. (2012) and Chai et al. (2019). An unexpectedly large number of *Sutorius* species were found and described in this study from Northern and Northeastern Thailand. Over a 7-year period (2012–2018), 26 *Sutorius* collections were obtained, of which 17 were obtained from northern Thailand (seven sites in three districts). These collections belonged to six novel and one known species. The other nine collections were from northeastern Thailand (collected from three sites in one district) and belonged to a single new species, different from those found in northern Thailand. Such a high diversity in a relatively small area, compared the single species known from the Americas and the single species known from Australia, was unexpected. It might be explained, however, by the location of the studied area in the Indo-Burma biodiversity hotspot. This hotspot is characterized by a wide variation in land form, climate, and latitude, which has led to the development of diverse natural habitats that support a high biodiversity (van Dijk et al., 2004). Moreover, the complex geological history associated with the Pleistocene climate oscillations has been suggested to produce the many localized centers of endemism observed in the hotspot (van Dijk et al., 2004). Northern Thailand, in particular, has a wide range of elevations, from around 300 m a.s.l. to 2,565 m a.s.l. at Doi Inthanon, the highest mountain in Thailand. This results in a diversity of forest types, many of which are dominated by ectomycorrhizal trees belonging to the Dipterocarpaceae, Fagaceae, Betulaceae, or Pinaceae. The center of diversity of *Sutorius* thus appears to be located in the studied area or possibly more broadly in the Greater Maekong Subregion. This region has been shown to shelter a high diversity of fungi, many of which only recently described or still unknown to science (e.g., Hyde et al., 2018; Thongbai et al., 2018). More studies on *Sutorius* are, however, needed in order to better document the distribution of this genus and clearly understand its biogeographic structure.

## Data Availability Statement

The datasets presented in this study can be found in online repositories. The names of the repository/repositories and accession number(s) can be found in the article/Supplementary Material.

## Author Contributions

OR, SV, and SL conceived and designed the study. SV, OR, and MA analyzed the data. SV, OR, and RH collected the specimens. SV and OR performed the experiments and wrote the manuscript. All authors reviewed and edited the manuscript.

## Conflict of Interest

The authors declare that the research was conducted in the absence of any commercial or financial relationships that could be construed as a potential conflict of interest.
