# Supplementary material for: An Unexpectedly High Number of New Sutorius (Boletaceae) Species From Northern and Northeastern Thailand
Source: Front Microbiol. 2021 Apr 12;12:643505. doi: 10.3389/fmicb.2021.643505 (PMC8072293; doi:10.3389/fmicb.2021.643505)
Supplement: Supplementary file 1 [file Data_Sheet_1.DOCX]

# Supplementary Figures

**Supplementary Figure 1.** Phylogenetic tree inferred from the *atp*6 dataset, of *Sutorius* species and selected Boletaceae in *Pulveroboletus* group, using Maximum Likelihood and Bayesian Inference methods (ML tree is presented). The three *Butyriboletus* species were used as outgroup. Bootstrap support values (BS ≥ 70%) and posterior probabilities (PP ≥ 0.90) are shown above the supported branches. The star (*) indicates additional collections with exactly identical sequences or sequences differing only by heteromorphisms (with the number of heteromorphisms mentioned in square brackets []).

**Supplementary Figure 2.** Phylogenetic tree inferred from the *tef*1 exons dataset, of *Sutorius* species and selected Boletaceae in *Pulveroboletus* group, using Maximum Likelihood and Bayesian Inference methods (ML tree is presented). The three *Butyriboletus* species were used as outgroup. Bootstrap support values (BS ≥ 70%) and posterior probabilities (PP ≥ 0.90) are shown above the supported branches. The star (*) indicates additional collections with exactly identical sequences or sequences differing only by heteromorphisms (with the number of heteromorphisms mentioned in square brackets []).

**Supplementary Figure 3.** Phylogenetic tree inferred from the *rpb*2 exons dataset, of *Sutorius* species and selected Boletaceae in *Pulveroboletus* group, using Maximum Likelihood and Bayesian Inference methods (ML tree is presented). The three *Butyriboletus* species were used as outgroup. Bootstrap support values (BS ≥ 70%) and posterior probabilities (PP ≥ 0.90) are shown above the supported branches. The star (*) indicates additional collections with exactly identical sequences or sequences differing only by heteromorphisms (with the number of heteromorphisms mentioned in square brackets []).

**Supplementary Figure 4.** Phylogenetic tree inferred from the introns of *tef*1 and intron of *rpb*2, of *Sutorius* species and selected Boletaceae in *Pulveroboletus* group, using Maximum Likelihood and Bayesian Inference methods (ML tree is presented). The three *Butyriboletus* species were used as outgroup. Bootstrap support values (BS ≥ 70%) and posterior probabilities (PP ≥ 0.90) are shown above the supported branches. The star (*) indicates additional collections with exactly identical sequences or sequences differing only by heteromorphisms (with the number of heteromorphisms mentioned in square brackets []).

**Supplementary Figure 5.** Phylogenetic tree inferred from the *atp*6, *tef*1 exons and *rpb*2 exons, of *Sutorius* species and selected Boletaceae in *Pulveroboletus* group, using Maximum Likelihood and Bayesian Inference methods (ML tree is presented). The three *Butyriboletus* species were used as outgroup. Bootstrap support values (BS ≥ 70%) and posterior probabilities (PP ≥ 0.90) are shown above the supported branches. The star (*) indicates additional collections with exactly identical sequences or sequences differing only by heteromorphisms (with the number of heteromorphisms mentioned in square brackets []).

**
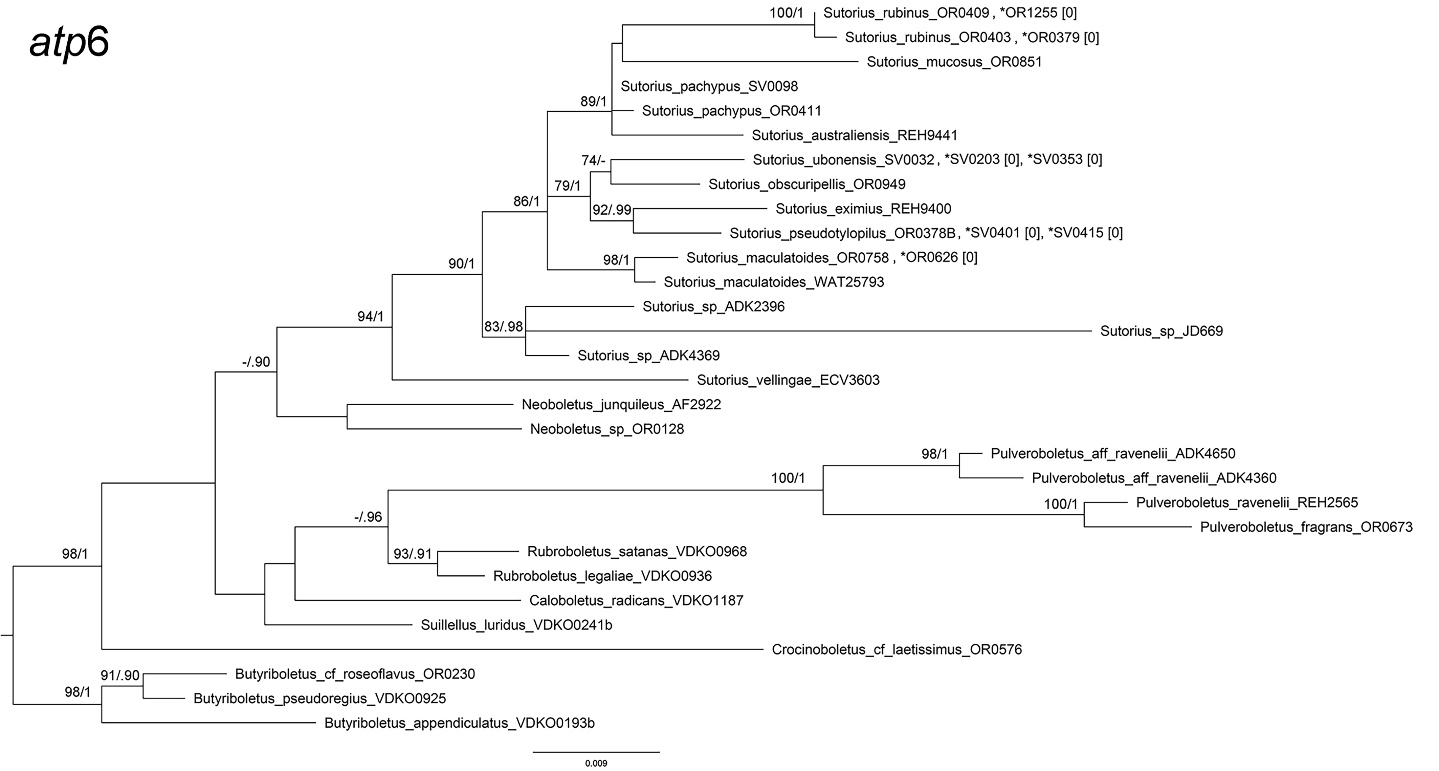
**

**Supplementary Figure 1.** Phylogenetic tree inferred from the *atp*6 dataset, of *Sutorius* species and selected Boletaceae in *Pulveroboletus* group, using Maximum Likelihood and Bayesian Inference methods (ML bipartition tree is presented). The three *Butyriboletus* species were used as outgroup. Bootstrap support values (BS ≥ 70%) and posterior probabilities (PP ≥ 0.90) are shown above the supported branches. The star (*) indicates additional collections with exactly identical sequences or sequences differing only by heteromorphisms (with the number of heteromorphisms mentioned in square brackets []).

**
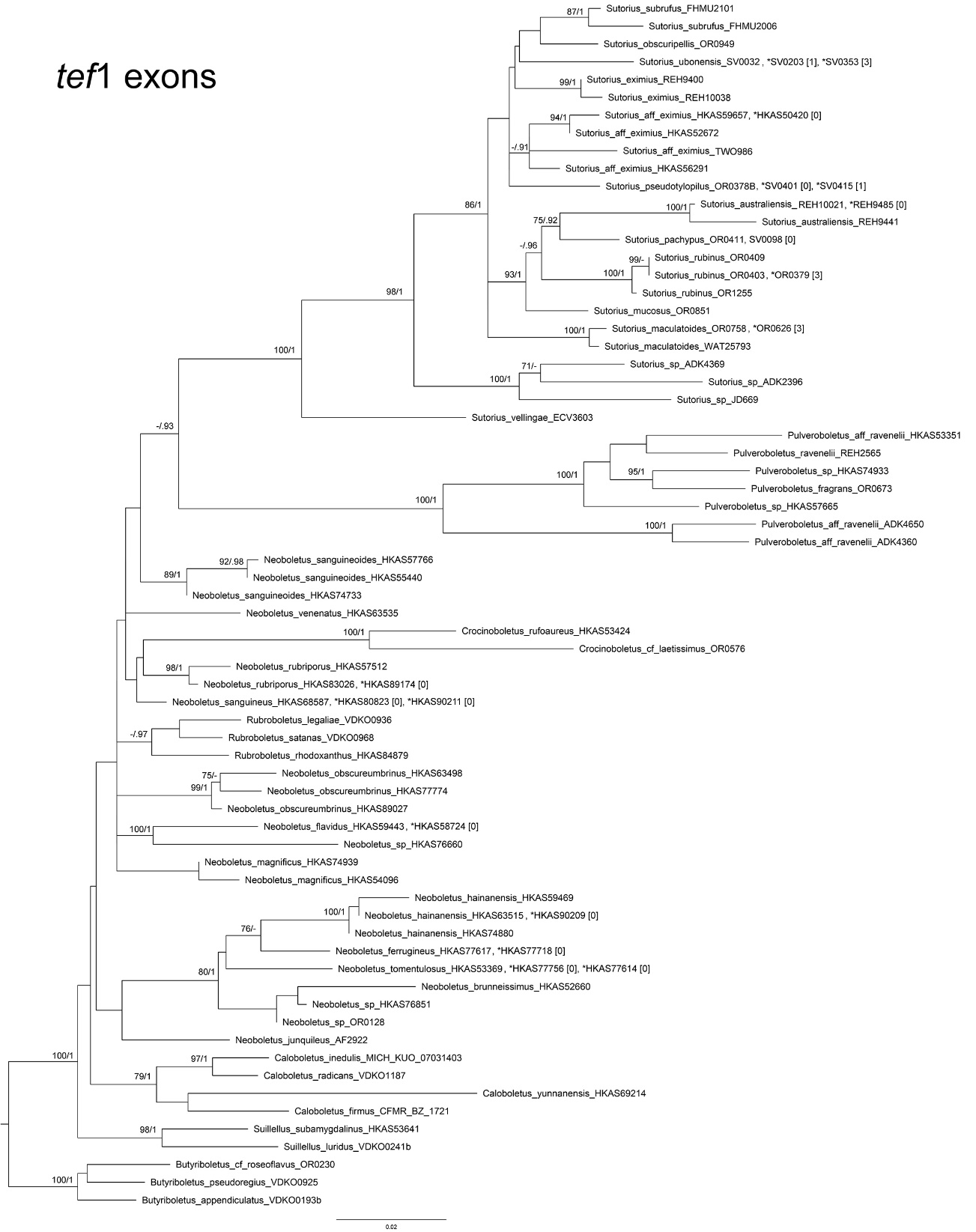
**

**Supplementary Figure 2.** Phylogenetic tree inferred from the *tef*1 exons dataset, of *Sutorius* species and selected Boletaceae in *Pulveroboletus* group, using Maximum Likelihood and Bayesian Inference methods (ML bipartition tree is presented). The three *Butyriboletus* species were used as outgroup. Bootstrap support values (BS ≥ 70%) and posterior probabilities (PP ≥ 0.90) are shown above the supported branches. The star (*) indicates additional collections with exactly identical sequences or sequences differing only by heteromorphisms (with the number of heteromorphisms mentioned in square brackets []).

**
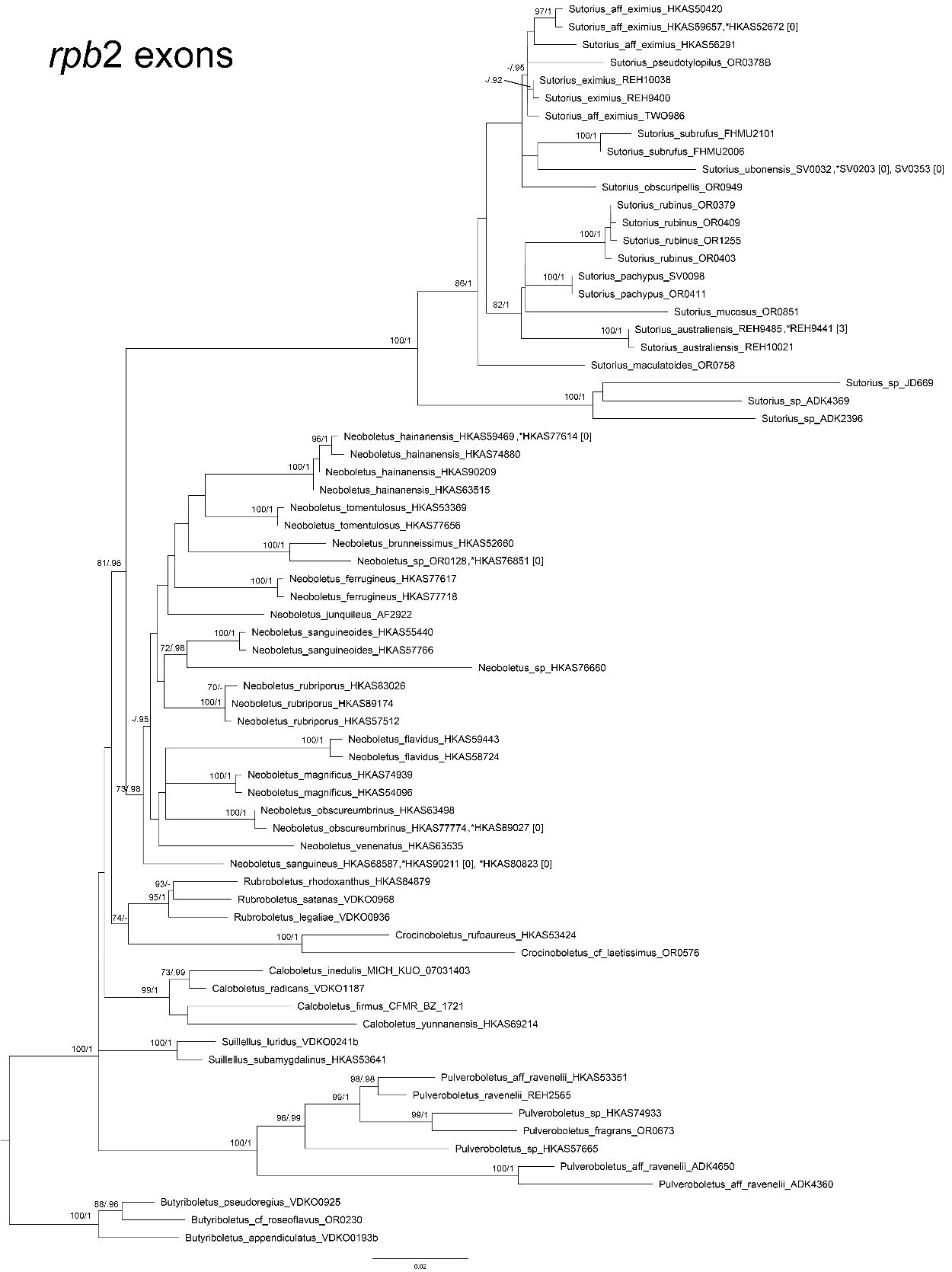
**

**Supplementary Figure 3.** Phylogenetic tree inferred from the *rpb*2 exons dataset, of *Sutorius* species and selected Boletaceae in *Pulveroboletus* group, using Maximum Likelihood and Bayesian Inference methods (ML bipartition tree is presented). The three *Butyriboletus* species were used as outgroup. Bootstrap support values (BS ≥ 70%) and posterior probabilities (PP ≥ 0.90) are shown above the supported branches. The star (*) indicates additional collections with exactly identical sequences or sequences differing only by heteromorphisms (with the number of heteromorphisms mentioned in square brackets []).

**
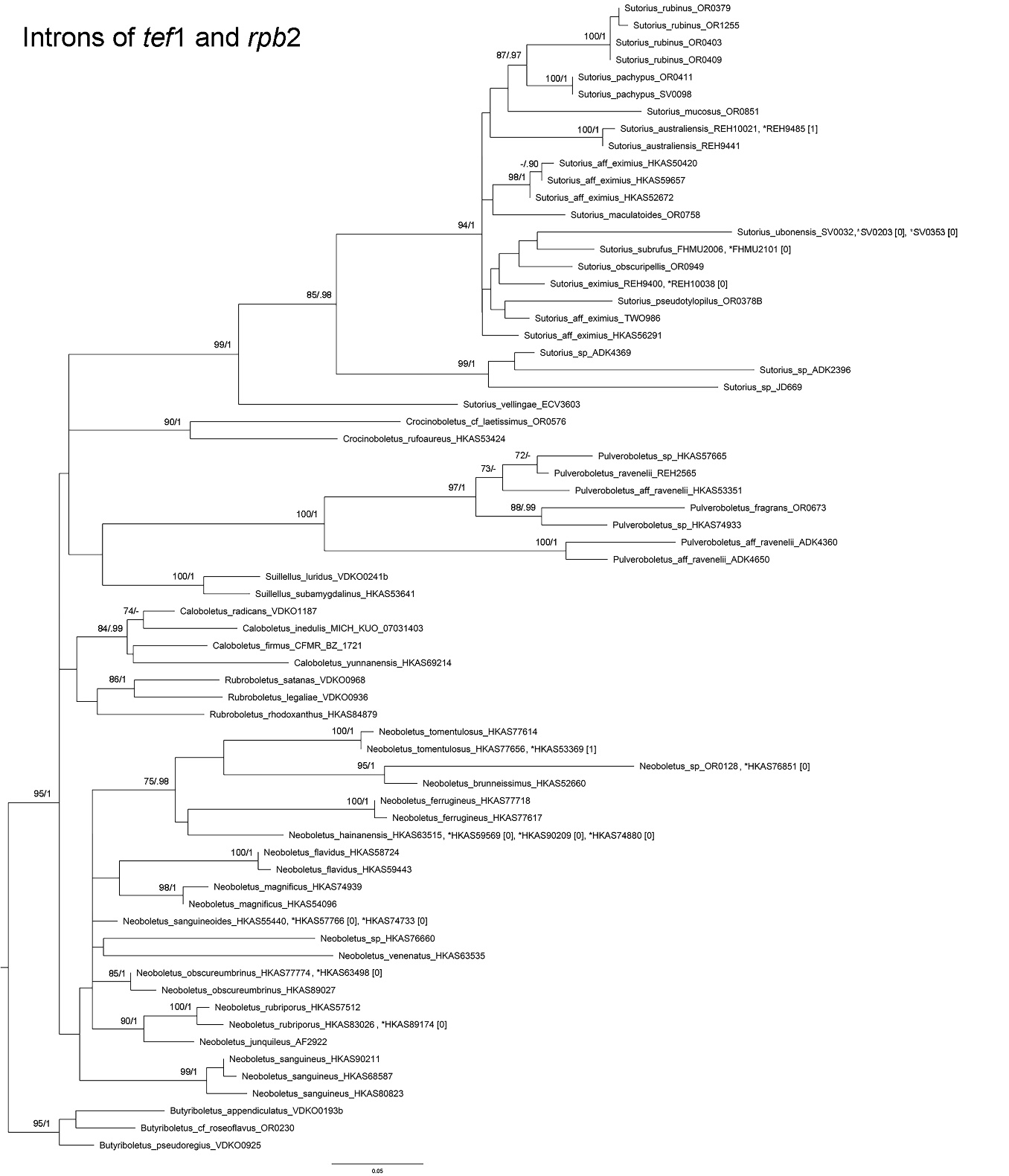
**

**Supplementary Figure 4.** Phylogenetic tree inferred from the introns of *tef*1 and intron of *rpb*2, of *Sutorius* species and selected Boletaceae in *Pulveroboletus* group, using Maximum Likelihood and Bayesian Inference methods (ML bipartition tree is presented). The three *Butyriboletus* species were used as outgroup. Bootstrap support values (BS ≥ 70%) and posterior probabilities (PP ≥ 0.90) are shown above the supported branches. The star (*) indicates additional collections with exactly identical sequences or sequences differing only by heteromorphisms (with the number of heteromorphisms mentioned in square brackets []).

**
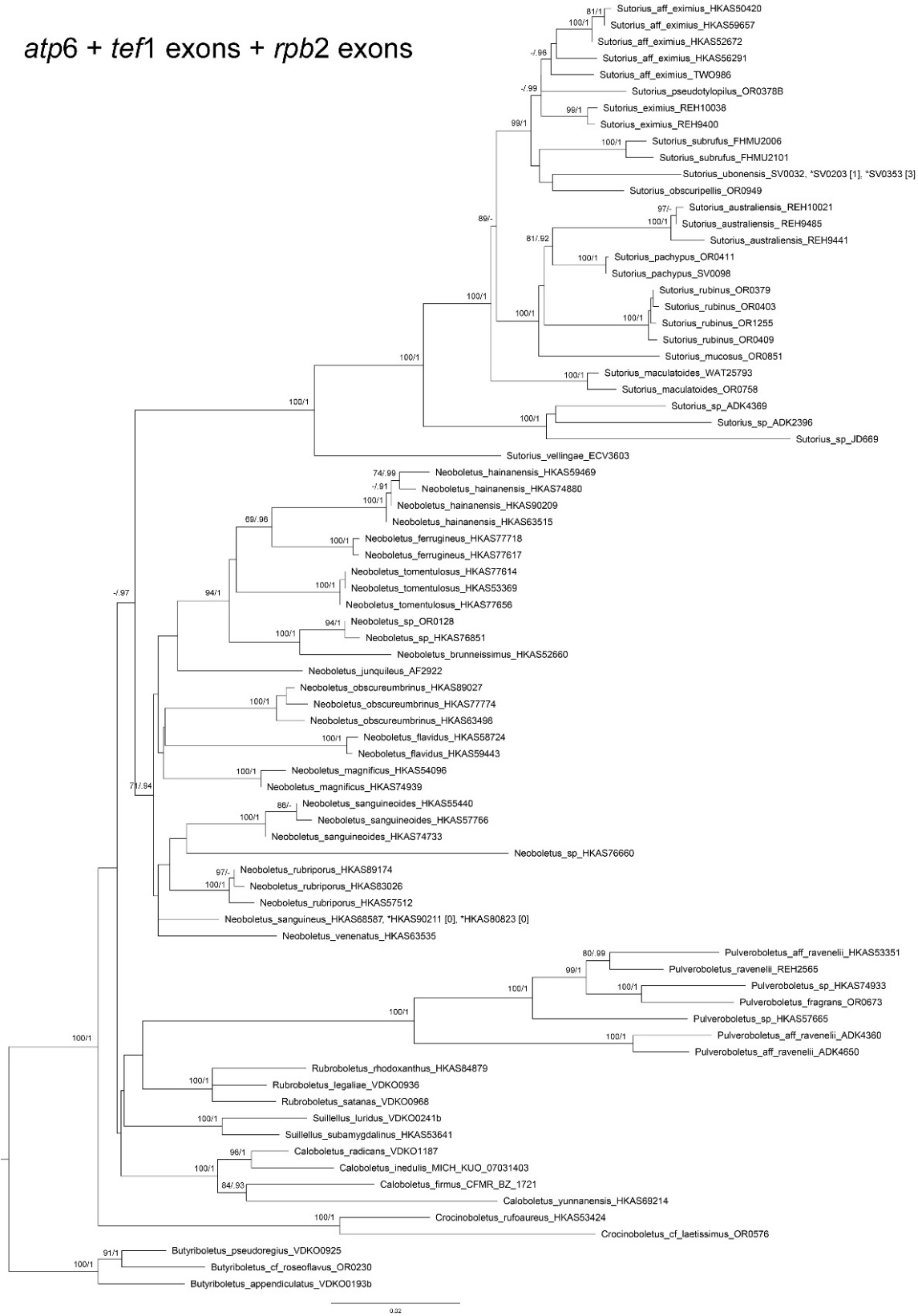
**

**Supplementary Figure 5.** Phylogenetic tree inferred from the *atp*6, *tef*1 exons and *rpb*2 exons, of *Sutorius* species and selected Boletaceae in *Pulveroboletus* group, using Maximum Likelihood and Bayesian Inference methods (ML bipartition tree is presented). The three *Butyriboletus* species were used as outgroup. Bootstrap support values (BS ≥ 70%) and posterior probabilities (PP ≥ 0.90) are shown above the supported branches. The star (*) indicates additional collections with exactly identical sequences or sequences differing only by heteromorphisms (with the number of heteromorphisms mentioned in square brackets []).
